# Phylogenic analysis of adhesion related genes *Mad1* revealed a positive selection for the evolution of trapping devices of nematode-trapping fungi

**DOI:** 10.1038/srep22609

**Published:** 2016-03-04

**Authors:** Juan Li, Yue Liu, Hongyan Zhu, Ke-Qin Zhang

**Affiliations:** 1 Laboratory for Conservation and Utilization of Bio-resources, and Key Laboratory for Microbial Resources of the Ministry of Education, Yunnan University, Kunming, 650091, P.R. China

## Abstract

Adhesions, the major components of the extracellular fibrillar polymers which accumulate on the outer surface of adhesive traps of nematode-trapping fungi, are thought to have played important roles during the evolution of trapping devices. Phylogenetic analyses based on the genes related to adhesive materials can be of great importance for understanding the evolution of trapping devices. Recently, *AoMad1,* one homologous gene of the entomopathogenic fungus *Metarhizium anisopliae* cell wall protein MAD1, has been functionally characterized as involved in the production of adhesions in the nematode-trapping fungus *Arthrobotrys oligospora*. In this study, we cloned *Mad1* homologous genes from nematode-trapping fungi with various trapping devices. Phylogenetic analyses suggested that species which formed nonadhesive constricting ring (CR) traps more basally placed and species with adhesive traps evolved along two lineages. Likelihood ratio tests (LRT) revealed that significant positive selective pressure likely acted on the ancestral trapping devices including both adhesive and mechanical traps, indicating that the *Mad1* genes likely played important roles during the evolution of nematode-trapping fungi. Our study provides new insights into the evolution of trapping devices of nematode-trapping fungi and also contributes to understanding the importance of adhesions during the evolution of nematode-trapping fungi.

Nematode-trapping fungi, a monophyletic group belonging to the order *Orbiliales* in Ascomycota, have evolved sophisticated hyphal structures (traps) such as adhesive networks (AN), adhesive knobs (AK) or adhesive columns (AC), nonconstricting rings (NCR) and constricting rings (CR) to capture nematodes^1^,^2^,^3^. This group of fungi has been proposed as potential biological control agents for controlling harmful plant-parasitic nematodes^4^,^5^,^6^,^7^,^8^. Also, many opportunistic pathogenic fungi can live both as a saprophyte and parasite to adapt to various ecosystems. The ability to switch between saprophytic and parasitic lifestyle is thus one of the most fundamental life strategies for fungi and also a key point for understanding their pathogenicity^8^. However, for most opportunistic pathogenic fungi, it is difficult to define their key time points of lifestyle-switching, which complicates understanding the pathogenesis mechanism^9^,^10^. Therefore, nematode-trapping fungi are considered a good model for understanding the pathogenesis mechanisms of fungi because trap formation is considered a key indicator for nematode-trapping fungi switching their lifestyles from saprophytic to predacious^11^.

Large morphologic variations have been observed among the trapping structures produced by nematode-trapping fungi^8^. Adhesive networks (AN) consists of complex three-dimensional nets, while adhesive columns (AC) is an erect branch. Adhesive knobs (AK) can be divided into stalked knobs and sessile knobs: stalked knobs are morphologically distinct globose structures which often are produced on the apex of a slender hyphal stalk, while sessile knobs are sessile on the hypha^3^,^7^. A layer of adhesive polymers is accumulated outside the cell wall of AN, AC and AK. These adhesive polymers are thought to be important materials which allow the fungi to adhere to the nematode cuticle^12^,^13^. Constricting rings (CR) is a ring formed by three cells. When a nematode enters into this trap, the three ring cells are triggered to swell rapidly and close around the nematode^14^,^15^,^16^. Therefore, the CR-forming species capture nematodes via mechanical forces^16^. These distinct trapping devices represent remarkable adaptations during fungal evolution^8^.

Previously, nematode-trapping fungi were classified into a number of genera based on the morphology of conidia and conidiophores but without consideration of trapping devices^17^,^18^. However, with the development of molecular methods, many studies suggested that trapping structures are more informative in generic delimitation among these fungi^2^,^19^,^20^,^21^. Accordingly, nematode-trapping fungi have been classified into three genera: *Arthrobotrys* is characterized by AN, *Dactylellina* by AK and/or NCR, and *Drechslerella* by CR^22^. It is noteworthy that those species which show similar morphology to nematode-trapping fungi but do not produce trap devices have been classified into genus *Dactylella* and are considered to be the ancestral species of nematode-trapping fungi^23^,^24^.

Trapping devices are significant for the survival of nematode-trapping fungi. At present, various hypotheses on the evolution of trapping devices have been proposed based on the phylogenetic analyses of several housekeeping genes^25^,^26^,^27^. Based on the phylogenetic analyses of 28S rDNA, 5.8S rDNA and β-tubulin genes, Li *et al.*^25^ proposed that AK is the ancestral type of trapping device which then evolved along two pathways: one way retained the adhesive material to form simple two-dimensional networks (AC), eventually forming complex three-dimension networks (AN); the other way lost the adhesive materials to form CR with three inflatable cells^25^. In addition, based on several molecular markers, such as RNA polymerase II subunit gene *rpb2*, elongation factor 1-α gene *ef1-*α, ß tubulin gene *bt* and the internal transcribed spacer region ITS, Yang *et al.* 2008^26^ suggested that trapping structures evolved along two lineages, yielding two distinct trapping mechanisms: one developed into CR and the other developed into adhesive traps. Among adhesive trapping devices, AN evolved from the others early and AK evolved through stalk elongation, with a final development of NCR^26^,^27^. Although conflicts exist between these evolutionary hypotheses, both of them hold that adhesive materials played important roles during the evolution of trapping devices. Thus, phylogenetic analyses of genes coding for adhesive proteins could improve understanding the evolution of trap devices.

Adhesive materials, the major components of the extracellular fibrillar polymers which are present on the outer surface of adhesive traps, are thought to enable the mycelia to adhere to nematodes and also serve as important constituents of the extracellular matrix that harbors many secreted virulence-related proteins^13^,^28^. To date, little is known about the exact components of adhesive materials located on the traps in nematode-trapping fungi. Recently, one cell wall protein MAD1 was characterized from the entomopathogenic fungus *Metarhizium anisopliae*^29^. The disruption of *Mad1* in *M. anisopliae* delayed germination, suppressed blastospore formation, and greatly reduced virulence to caterpillars^29^. Moreover, one homolog of *Mad1*, *AoMad1,* has been identified and functionally studied in the nematode-trapping fungus *A. oligospora*. Transmission electron microscopic (TEM) investigation found that almost all the surface polymers were absent from the Δ*AoMad1* cell wall, suggesting that *AoMad1* is involved in the production of adhesive proteins in nematode-trapping fungi^30^.

At present, three whole genomes of the AN-forming species *A. oligospora*, the AK and NCR-forming species *Dactylellina haptotyla* (also known as *Monacrosporium haptotylum*) and the CR-forming species *Drechslerella stenobrocha*) have been sequenced^11^,^31^,^32^, which provides a good opportunity to design degenerate primers to clone *Mad1* homologs from different nematode-trapping fungi. In this study, we cloned *Mad1* homologs from nematode-trapping fungi with various trapping devices and the species belonging to genus **Dactylella**. We hypothesize that *Mad1* encoding genes may play important roles during the evolution of trap devices in nematode-trapping species. To accomplish this, phylogenetic analyses based on 47 *Mad1* homologs were performed in this study including 44 genes newly cloned in this study and three genes from the three whole genome sequenced fungi. Also, the possible selection pressures responsible for *Mad1* genes in nematode-trapping species were investigated. Our study provides new insights into the evolution of trap devices based on the genes related to adhesive materials.

## Materials and Methods

### Microorganisms and DNA extraction

The 45 fungal strains used in this study (Table 1) are permanently stored in the Yunnan Microbiological Fermentation Culture Collection Center (YMF). Fungi were cultured on PDA medium at 28 °C for 8–15 day. Their mycelia were scraped off from the plate then collected and genomic DNA was isolated from about 200mg mycelia using the E.Z.N.A.^@^ Fungal DNA Mini kits (Omega Bio-Tek, Inc. USA) following the manufacturer’s protocol.

### Primer design and cloning of *Mad1* homologs

Degenerate primers (*Mad1F*: 5′-TACAGTG(C/T)GGTGGAGCCAAGAG-3′ and *Mad1R*:5′-CTT(G/A)ACTGGGCAGACGGTGAC-3′) were designed using DNAman software package (Version 5.2.2, Lynnon Biosoft, Canada) based on the homologs of *Mad1* from the three whole genome sequenced nematode-trapping fungi (GenBank numbers XM_011114756 in *D. haptotyla*, XM_011123119 in *A. oligospora*, and KI966443 in *D. stenobrocha*) and used to amplify the gene fragments of *Mad1* homologs from those species employed in this study. The PCR reaction mixture consisted of 0.5 μL Taq DNA polymerase, 5 μL of reaction mixture buffer, 3 μL of 25 mM MgCl_2_, 1 μL of 2.5 mM dNTPs, 1 μL of 100 μM degenerate primers, and 0.5–1.0 μg quantified DNA template in a final volume of 50 μL supplied with double-distilled sterile water. Amplification started at 95 °C for 5 min, followed by 35 cycles with 95 °C for 40 s, 51 °C for 40 s, and 72 °C for 1.5 min. After the last cycle, the reaction mixture was maintained at 72 °C for 10 min for a final extension step. The universal primers (ITS4: 5′-TCCTCCGCTTATTGATATGC-3′ and ITS5:5′-GGAAGTAAAAGTCGTAACAAGG-3′) were also used to clone the ITS sequences from the fungi species in this study for genotyping purposes^33^.

### Sequencing and analysis

Amplified PCR products were electrophoresed on 1% agarose gels and purified using the DNA fragment purification kit version 2.0 (Takara, Japan) and then sequenced on an ABI 3730 automated sequencer in both directions using the same PCR primers (Perkin-Elmer, USA). Sequence assembly was performed using the SeqMan software (DNA Star software package, DNASTAR, Inc. USA) and DNAman software package (Version 5.2.2, Lynnon Biosoft, Canada). Conserved protein domains of *Mad1* were identified using InterProScan (http://www.ebi.ac.uk/Tools/pfa/iprscan/) with default parameter settings^34^.

### Phylogenetic analysis

Codon-based nucleotide alignment was generated by using MUSCLE v3.5 with default settings^35^. The ambiguous areas of alignment were removed by using the program Gblocks 0.91b with default parameters with the exception that the gap selection criterion “with half” was used^22^,^36^. An alignment consisting of 1272-bp alignment (corresponding to 440 amino acids) was obtained (Supplementary Fig. S1). ITS sequences of the nematode-trapping fungi were also aligned by MUSCLE v3.5^35^ and the ambiguous areas were also removed by Gblocks 0.91b with default parameters^22^,^36^. Finally, a total of 502-bp alignment was obtained.

Three tree-building methods were performed for phylogenetic reconstructions of *Mad1* genes. The program MEGA 6^37^ was used to construct a neighbor joining (NJ) tree, and MrBayes 3.1.2^38^ was used to perform Bayesian analysis. The Maximum Likelihood (ML) analysis was performed using PHYML 3.0^39^. In the NJ analysis, pairwise deletion option for gaps was used. In the ML analysis, the model GTR+I+G of sequence evolution was chosen by using Akaike information criterion as implemented in Modeltest version 3.7^40^. The reliability of these tree topologies was evaluated using bootstrap support^41^ with 1000 replicates for NJ and 100 for ML analysis. The parameters estimated by Modeltest were also used in the priors of Bayesian inference with MrBayes version 3.1.2^38^. Bayesian analysis started with randomly generated trees and Metropolis-coupled Markov chain Monte Carlo (MCMC) analyses were run for 2 × 10^6^ generations. The run was stopped when the average standard deviation of split frequencies was less than 0.01 in all cases (MrBayes 3.1.2 manual). To ensure that these analyses were not trapped in local optima, the dataset was run three times independently. Bayesian posterior probabilities (PP) from the 50% majority-rule consensus tree were calculated to provide the estimates of nodal support in Bayesian phylogenies. For the ITS sequences of the nematode-trapping fungi in this study, only ML tree was produced using PHYML 3.0^38^. The best-fitting model GTR+I+G estimated by program Modeltest version 3.7^39^ was used in the ML analysis. The reliability of the tree topology was evaluated using bootstrap support^40^ with 100 replications.

### Selective pressures analyses

**Figure 1:**
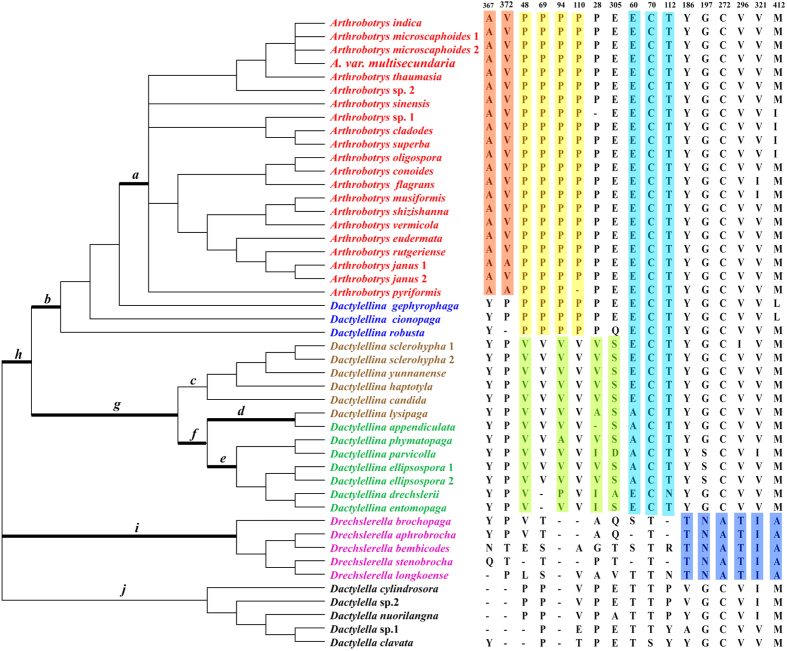
Phylogenetic tree of *Mad1* genes used for codon-based maximum likelihood analysis in PAML. Phylogenetic trees with inconsistent nodes from different tree-building methods and poor statistical (BS value < 70) support were collapsed into polytomy. Branch-site model tests were performed for the ancestral branches (*a*–*i*) of each type of trap structure. The branches with significant evidence of positive selection are indicated with a thick line. The putative positively selected residues along different branches are shaded with different colors.

The ratio ω (*dN/dS*) is the ratio of the number of non-synonymous substitutions per non-synonymous site (*dN*) to the number of synonymous substitutions per synonymous site (*dS*), which provides an indication of the change in selective pressures^42^. *dN/dS* ratios of 1, <1, and >1 are indicative of neutral evolution, purifying selection, and positive selection on the protein involved, respectively^43^,^44^. To investigate the possible selective forces behind *Mad1* homologs in nematode-trapping fungi with various trapping structures, the codon substitution models implemented in the CODEML program in the PAML 4.4b package^45^ were used to analyze changes of selective pressure. Given that the likelihood may be sensitive to the tree topology used, inconsistent nodes from different tree-building methods and with poor statistical support were collapsed into a polytomy^46^. The collapsed tree (Fig. 1) was then used to conduct the analysis to determine the signatures of positive selection. Two branch-specific models were compared, i.e., the “one-ratio” (M0) model which assumes the same ω ratio for all branches was compared with the “free-ratios” model which assumes an independent ω ratio for each branch^47^. Secondly, site-specific models M1a, M2a, M7, and M8, which allow for variable selection patterns among amino acid sites, were used to test for the presence of sites under positive selection. M2a and M8 models allow for positively selected sites. When these two positive-selection models fitted the data significantly better than the corresponding null models (M1a and M8a), the presence of sites with ω > 1 was suggested. The conservative Empirical Bayes approach was then used to calculate the posterior probabilities of a specific codon site and identify those most likely to be under positive selection^48^. The “branch-site” model, which accommodates ω ratios to vary both among lineages of interest and amino acid sites, was also considered here^49^. We used branch-site Model A as a stringency test (test 2) and identified amino acid sites under positive selection by an empirical Bayes approach along the lineages of interest^49^,^50^. The log-likelihoods for the null and alternative models were used to calculate a likelihood ratio test (LRT) statistic, which was then compared against the χ2 distribution (with a critical value of 3.84 at a 5% significance level)^45^. In addition, the Bonferroni correction^51^,^52^ was also applied for multiple testing in the analysis according to the number of tests of significance performed.

## Results

### *Mad1* homologs from nematode-trapping fungi

Using the degenerate primers *Mad1F* and *Mad1R* to amplify the 3′ terminal fragments which contain the functional domains of *Mad1* homologs, 39 gene fragments ranging from 1193-bp to 1826-bp in length were amplified from their corresponding nematode-trapping fungi and 5 fragments were obtained from 5 *Dactylella* species (Table 1, GenBank nos: KT932011-KT932054). homologsA total of 47 fragments were used for subsequent analyses (Table 1). In addition, 34 ITS fragments of the corresponding 34 strains were amplified in our study (Table 1, GenBank nos: KT932055-KT932088) and 10 ITS sequences were downloaded from the NCBI database (Table 1). Finally, in total of 44 ITS fragments were used for phylogenetic analyses with the exception of those ITS sequences from the three strains: *Arthrobotrys rutgeriense*, *Arthrobotrys* sp. 1 and *Dactylellina ellipsospora* 1 were not obtained in our study.

**Figure 2:**
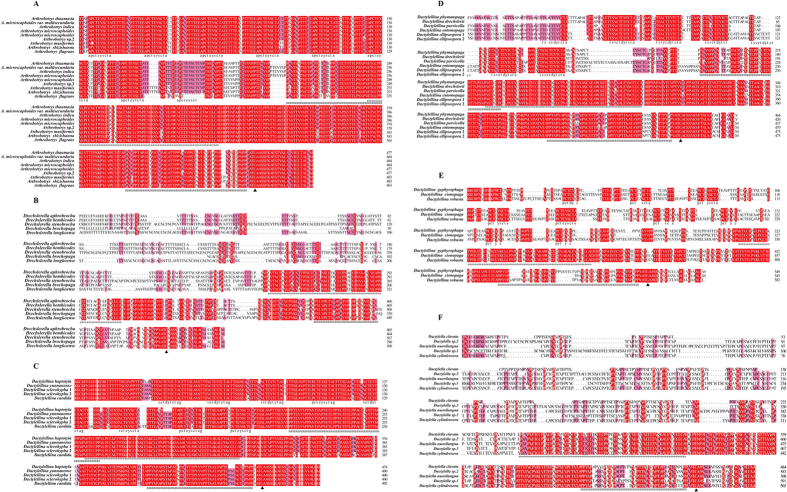
Protein alignment of *Mad1* genes in nematode-trapping fungi. (**A**) alignment of *Mad1* genes from representative AN-forming species. (**B**) alignment of *Mad1* genes from representative CR-forming species. (**C**) alignment of *Mad1* genes from representative AK and NCR-forming species. (**D**), alignment of *Mad1* genes from representative AK-forming species. (**E**) alignment of *Mad1* genes from representative AC-forming species. (**F**) alignment of *Mad1* genes from *Dactylella* species. Areas shaded in red are conserved regions (100% similarity). Areas shaded in pink have a high degree of homology (more than 75% similarity). *indicates the CFEM domain, ^#^indicates Pro-rich domain, ▲ indicates the GPI site.

Functional domain analyses suggested that the *Mad1* homologs in nematode-trapping fungi (Fig. 2) contain several domains similar to those in *M. anisopliae.* Intriguingly, independent alignment of translated amino acids shows that there are significant differences among the sequences of different trapping devices. As seen in Fig. 2, the *Mad1* homologs derived from those species forming adhesive traps are much more conserved than those genes from the mechanical CR-forming species. The most highly conserved *Mad1* genes from the AN-forming species contain a Threonine-rich (Thr-rich) domain composed of eight repeats of “EAPCTEYSCTA” and two Proline-rich (Pro-rich) domains (indicated by pound signs in Fig. 2) located at the two sides of a CFEM domain (indicated by asterisks in Fig. 2). Also, a glycosylphosphatidylinositol (GPI)-anchoring signal peptide was identified at their C-terminal ends (indicated by black triangle in Fig. 2). The *Mad1* genes cloned from those species which can form AK and NCR (Fig. 2C) are also composed of four functional domains: the Thr-rich domain consisting of eight repeats of “V/PCTD/EYCTAG”, the two Pro-rich domains at the two sides of the CFEM domain and the conserved GPI site, showing similar structures to those of genes from AN-forming species. The genes from the AK-forming species (Fig. 2D) are highly similar to the genes of AN and AK and NCR forming species with the exception that the repeated sequences are “TSVCTDYTCTA” and only seven repeats are found. Moreover, the genes from the AC-forming species show less conservation than other adhesive trap-forming species. There are many amino acid mutations at the repeat domains and only one Pro-rich domain is found on the right of the CEFM domain (Fig. 2E). Interestingly, for the genes from the CR-forming species and *Dactylella* species, no repeated sequences can be found at the N-terminal and only the CFEM domain and the GPI site are conserved. Moreover, at the right side of the CFEM domain, fewer fragments of the Pro-rich domain are found (Fig. 2B,F).

### Phylogenetic analyses

In our study, an alignment consisting of 1272 bp (corresponding to 424 amino acids) was obtained and used for subsequent analyses (Supplementary Fig. S1).

**Figure 3:**
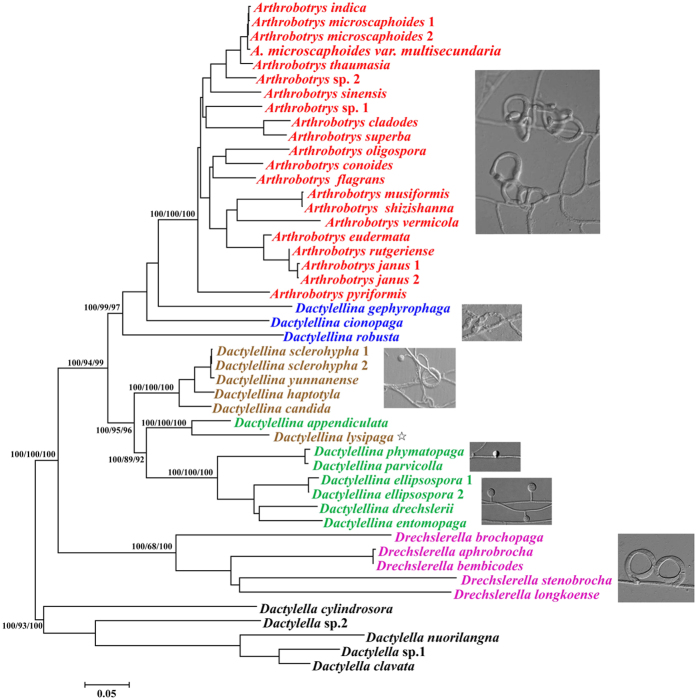
Phylogenetic analyses based on the encoding sequences of *Mad1* genes. Bayesian, maximum-likelihood (ML), and neighbor-joining (NJ) tree reconstructions of the *Mad1* gene sequences presented similar overall topologies. The bootstrap values of each branch for different methodologies are indicated (Bayesian/ML/NJ). The species names which can produce different trapping devices are showed with different colors: red for AN-forming species, blue for AC-forming species, brown for AK and NCR-forming species, green for AK-forming species, pink for CR-forming species and black for *Dactylella* species.

Phylogenetic analyses based on the fragments of *Mad1* homologs consistently revealed similar topologies with high bootstrap value or posterior probabilities (PP) (Fig. 3). Cladograms revealed that the species which form similar trapping structures were clustered into the same group/subgroup, implying distinctive signatures of different trapping devices. In our analysis, the species (pink color in Fig. 3) with nonadhesive CR traps more basally placed from species with adhesive traps (PP = 100% in Bayesian, BS = 68% in ML, BS = 100% in NJ). Subsequently, the adhesive traps resulted in two main clades: one clade (PP = 100% in Bayesian, BS = 99% in ML, BS = 97% in NJ) consists of species with AC (blue color in Fig. 3) and AN (red color in Fig. 3), and the species with AC more basally placed from other species with AN. The other clade (PP = 100% in Bayesian, BS = 95% in ML, BS = 96% in NJ) contains subclades corresponding to those species which can form both AK and NCR (brown color in Fig. 3) or those species only forming AK (green color in Fig. 3). Within this clade, the species forming AK associated with NCR separated early from other species, and one species forming AK and NCR showed close relationships with the species with AK. Moreover, two species forming sessile knobs (*Dactylellina parvicollis* and *Dactylellina phymatopaga*) diverged early from other species forming stalked knobs (*Dactylellina drechsleri*, *Dactylellina entomopaga*, and *Dactylellina ellipsospora*) (Fig. 3). However, phylogenetic trees based on the fragments of ITS (Supplementary Fig. S2) show similar topologies with the phylogenetic trees of *Mad1* fragments with the exception that the species with AC first diverged from other adhesive traps (Supplementary Fig. S3).

### Selective pressure analyses

To investigate the possible selective forces behind the *Mad1* homologs during the evolution of various trapping devices in nematode-trapping fungi, we conducted LRT for those ancestral branches of each type of trap structure. Table 2 shows the evidence for positive selection of *Mad1* genes. In the branch-specific model analyses, the free-ratio model, M1a, revealed a significantly better fit to the data than did the one-ratio model, M0 (2Δ*L* = 348.64117, p < 0.001, Table 2), suggesting that *Mad1*genes have been the subjects of different selective pressures. In the site-specific model analyses, although the LRT of M2a/M1a did not achieve statistical significance (2Δ*L* = 0, P = 1.000, Table 2), M8, another positive-selection model, provided a significantly better fit to the data than did the neutral model (M7) (2Δ*L* = 1992.17435, P < 0.001, Table 2), suggesting the possibility of positive selection acting on the *Mad1* genes in the nematode-trapping fungi examined here.

When we performed the branch-site model tests for those ancestral branches of each type of trap structure (10 branches in total, *a–j* as indicated in Fig. 1), we found that except for branches *c* and *j*, all branches (branches *a*, *b, d, e, f*, *g, h and i*) showed signs of positive selection (Fig. 1). After Bonferroni correction for multiple testing, we found that LRT results were still significant in eight branches (p <0.005) (Table 2, Fig. 1). Remarkably, several positively selected residues were also identified for these branches with high posterior probabilities (Table 2 and Fig. 1).

## Discussion

In examining the *Mad1* sequences from each type of trapping device, we found that all sequences contained a predicted glycosylphosphatidylinositol cell wall anchor site at their C-terminal region, implying they are cell wall proteins. The major differences among these sequences from different trap-forming groups are in the Thr-rich random repeats domain. Previous studies revealed that the tandem repeats are heavily glycosylated to produce a rigid elongated structure that holds the adhesive N-terminal domain at the cell surface^53^,^54^, and the tandem repeat region of the *C. albicans* cell wall protein FLO11 is required for yeast pseudohypha formation^55^. This region was shown to be necessary and sufficient for adhesion to tick cells^56^. Thus, the random repeats domain may be related to the adhesive properties of *Mad1*, which permit nematode-trapping fungi to adhere to nematode cuticles. As seen in Fig. 2, all the sequences amplified from the species which can form adhesive traps contain Thr-rich domains though the amino acid sequences and numbers of the tandem repeats are different among trap types, suggesting that the N-terminal ligand binding region located outside the cell surface may be different in various trapping structures. Especially, the Thr-rich regions of the *Mad1* genes from the AC-forming species are less conserved (Fig. 2), suggesting the less adhesive properties of the Mad1 proteins in AC-forming species. It is surprising that no tandem repeats are found in the sequences from CR-forming species (Fig. 2), suggesting that the N-terminal ligand binding region may be shorter or absent in the CR-forming species, consistent with the observation that the CR-forming species capture nematodes mainly using mechanical force.

Subsequently, phylogenetic analyses based on the *Mad1* homologs consistently suggested that the trap devices evolved in two ways with two different trapping mechanisms (adhesive and nonadhesive) (Fig. 3). The nonadhesive traps, CR, separated early from species with adhesive traps, suggesting the primitive character of CR. This result concurs with previous studies performed by Yang *et al.*^26^,^27^. Evolution of the adhesive trapping structures also separated in two directions: the evolution of AN from AC, and the evolution of species which only produce AK from the species producing AK associated with NCR. Within the latter direction, NCR were generally discarded during evolution because of their low efficiency in capturing nematodes. Also, the stalked knobs evolved from sessile knobs. Moreover, the phylogenetic tree (Supplementary Fig. S3) based on the ITS fragments of nematode-trapping fungi in our study shows limited differences from previous phylogenetic trees constructed based on the combined data from several housekeeping genes, especially the relationships among the adhesive traps. In conclusion, all the phylogenetic trees including those previously reported and ours in this study consistently supported the results that the adhesive and nonadhesive traps evolved independently, and the CR is the most ancestral trap of nematode-trapping fungi. However, obtaining more adhesive proteins or other genes related to trap formation may provide more information for understanding the evolution of trap structures. Our study based on the adhesive protein MAD1 proposed a new evolutionary hypothesis of the nematode-trapping fungi producing various trapping devices.

Interestingly, LRT analysis suggested that the *Mad1* genes most likely underwent positive selection during the evolution of nematode-trapping fungi (Fig. 1 and Table 2). It is reasonable to presume that significant selective pressures acted on the ancestral branches of adhesive trapping devices (branches *a*, *b*, *g*, and *h*) because MAD1 is secreted outside the cell wall to help nematode-trapping fungi to adhere to nematodes. To maintain their function, selective pressure might have acted on these lineages and promoted fungal adaptions. However, we did not observed positive selective pressure on the branch which produced AK and NCR (branch *c*). The ancestral branch (branch *g*) representing both AK and NCR-forming and AK-forming species experienced significant pressure, suggesting that positive selective pressures likely acted on them at the beginning. Interestingly, for the lineages of adhesive traps, most of the positively selected sites were located at the Thr-rich domains and some sites were even located within the random repeats (such as V48P, V69P, V94P, and V110P in branch *b*; P28V/A/I, P48V, and P94V in branch *g*), further implying the importance of the Thr-rich domain of *Mad1* genes in fungi with adhesive traps.

Surprisingly, significant positive selective pressure also likely acted on the ancestral branch of CR-forming species which do not use adhesions to capture nematodes (branch *i*). This unexpected result indicates *Mad1* genes likely have evolved other uncharacterized functions in CR-forming fungi. Recently, the *AoMad1* gene has been knocked out from the nematode-trapping fungus *A. oligospora*. Interestingly, although the cell surface adhesive materials within the network disappeared and the cell wall structure showed more porosity after deletion of the gene *AoMad1*, more traps were formed in the mutant than in the wild type with the presence of nematodes. Meanwhile, a great number of genes were differentially expressed by transcriptomic analysis. In view of this, Liang *et al.* assumed that *AoMad1* may play a key role in *A. oligospora*’s recognition of host signals and trigger life style switching. However, despite the lack of molecular experiments on CR-forming nematode-trapping fungi, we propose that the *Mad1* genes may have multiple functions in nematode-trapping fungi beyond allowing fungi to adhere to nematodes. In addition, the selected positive sites on this branch were mainly located on the CFEM domain and Pro-rich domain (Y186T, G197N, C272A, V296T, V321I, and M412A), suggesting that these domains are very important for *Mad1* genes to execute their functions in CR-forming fungi. Also, as the domains are conserved in the other nematode-trapping species, we speculate that the Thr-rich domain likely plays a key role in helping nematode-trapping fungi capture nematodes, while the CEFM domain and Pro-rich domain may play other functions in nematode-trapping fungi.

## Conclusions

Based on the phylogenetic analyses of the *Mad1* fragments related to adhesive materials, our study provides new insights into the evolution of trapping devices of nematode-trapping fungi. As with the evolutionary hypothesis proposed based on the housekeeping rDNA genes, our phylogenetic analyses provide evidence that the adhesive and nonadhesive traps evolved independently and the CR is the most ancestral type of trap in nematode-trapping fungi. However, there are differences among the evolutionary paths leading to different adhesive traps. Analyses based on more functional genes related to trap formation may provide more information for understanding the evolution of trap structures.

In addition, the evidence for positive selection detected in the *Mad1* genes of nematode-trapping fungi in the present study suggests that the *Mad1* genes may have played important roles during the evolution of nematode-trapping fungi. Also, it will be interesting to test the functional effects of amino acid substitutions for the identified positively selected sites in future studies.

## Additional Information

**How to cite this article**: Li, J. *et al.* Phylogenic analysis of adhesion related genes *Mad1* revealed a positive selection for the evolution of trapping devices of nematode-trapping fungi. *Sci. Rep.* **6**, 22609; doi: 10.1038/srep22609 (2016).

## Supplementary Material

Supplementary Information

## Figures and Tables

**Table 1:** GenBank accession numbers for sequences used in the phylogenetic analysis.

Species names	Strain number in our study	Trap devices	GenBank Nos of Mad1	Length of Mad1(bp)	GenBank Nos of ITS
*Dactylellina gephyrophaga*	YMF1.00033	AC	KT932031	1649	KT932061
*Dactylellina cionopaga*	YMF1.00569	AC	KT932032	1637	AY944137
*Dactylellina robusta*	YMF1.01413	AC	KT932033	1751	DQ999821
*Dactylellina parvicolla*	YMF1.00029	AK	KT932043	1430	KT932059
*Dactylellina ellipsospora 1*	YMF1.00032	AK	KT932038	1406	/*
*Dactylellina drechslerii*	YMF1.00116	AK	KT932040	1316	KT932078
*Dactylellina appendiculata*	YMF1.01465	AK	KT932044	1502	KT932084
*Dactylellina entomopaga*	YMF1.01467	AK	KT932041	1424	AY965758
*Dactylellina phymatopaga*	YMF1.01474	AK	KT932042	1436	KT932060
*Dactylellina ellipsospora 2*	YMF1.01853	AK	KT932039	1283	KT932063
*Dactylellina sclerohypha 1*	YMF1.00041	AK$NCR	KT932036	1472	KT932062
*Dactylellina lysipaga*	YMF1.00535	AK$NCR	KT932045	1508	KT932082
*Dactylellina sclerohypha 2*	YMF1.00540	AK$NCR	KT932035	1472	KT932066
*Dactylellina candida*	YMF1.00543	AK$NCR	KT932037	1478	KT932067
*Dactylellina yunnanense*	YMF1.01466	AK$NCR	KT932034	1472	KT932076
*Dactylellina haptotyla*	/**	AK$NCR	XM_011114756	1992	AF106523
*Arthrobotrys conoides*	YMF1.00009	AN	KT932025	1613	KT932055
*Arthrobotrys superba*	YMF1.00016	AN	KT932030	1487	U51949
*Arthrobotrys pyriformis*	YMF1.00018	AN	KT932028	1619	KT932056
*Arthrobotrys shizishanna*	YMF1.00022	AN	KT932024	1460	KT932088
*Arthrobotrys sinensis*	YMF1.00025	AN	KT932017	1544	KT932069
*Arthrobotrys microscaphoides* 1	YMF1.00028	AN	KT932014	1532	KT932058
*Arthrobotrys rutgeriense*	YMF1.00040	AN	KT932021	1463	/
*Arthrobotrys vermicola*	YMF1.00534	AN	KT932022	1511	KT932065
*Arthrobotrys eudermata*	YMF1.00545	AN	KT932018	1345	KT932087
*Arthrobotrys* sp. 1	YMF1.01425	AN	KT932027	1601	/
*Arthrobotrys microscaphoides 2*	YMF1.00546	AN	KT932015	1511	KT932057
*Arthrobotrys* sp. 2	YMF1.00547	AN	KT932016	1511	KT932070
*Arthrobotrys musiformis*	YMF1.00575	AN	KT932023	1460	KT932072
*Arthrobotrys janus 1*	YMF1.01312	AN	KT932019	1484	KT932074
*Arthrobotrys flagrans*	YMF1.01471	AN	KT932026	1472	KT932085
*A. microscaphoides var. multisecundaria*	YMF1.01821	AN	KT932012	1532	KT932077
*Arthrobotrys indica*	YMF1.01845	AN	KT932013	1532	KT932086
*Arthrobotrys janus 2*	YMF1.01889	AN	KT932020	1484	KT932068
*Arthrobotrys cladodes*	YMF1.03233	AN	KT932029	1589	U51945
*Arthrobotrys thaumasia*	YMF1.03502	AN	KT932011	1511	KT932081
*Arthrobotrys oligospora*	/	AN	XM_011123119	2157	KJ938573
*Drechslerella bembicodes*	YMF1.01429	CR	KT932047	1391	KT932075
*Drechslerella brochopaga*	YMF1.01829	CR	KT932048	1193	FJ380936
*Drechslerella longkoense*	YMF1.01863	CR	KT932049	1826	KT932079
*Drechslerella aphrobrocha*	YMF1.01881	CR	KT932046	1394	KT932080
*Drechslerella stenobrocha*	/	CR	KI966443	2229	AY773460
*Dactylella clavata*	YMF1.00124	None	KT932051	1805	KT932064
*Dactylella* sp.2	YMF1.00568	None	KT932053	1454	KT932071
*Dactylella nuorilangna*	YMF1.00582	None	KT932052	1760	KT932073
*Dactylella* sp.1	YMF1.01463	None	KT932050	1706	KT932083
*Dactylella cylindrosora*	YMF1.03528	None	KT932054	1451	AF106538

^*^The sequences did not obtained based on primers ITS4 and ITS5.

^**^The three species were whole genome sequenced.

**Table 2:**
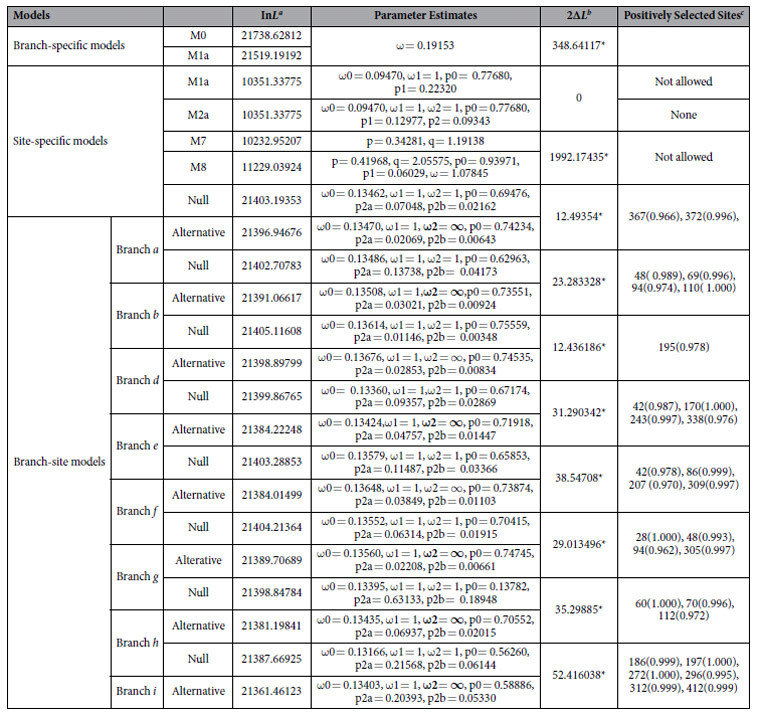
CODEML analyses of selective pressures for Mad1 genes in nematode-trapping fungi.

^a^In*L* is the log-likelihood scores.

^b^LRT to detect adaptive evolution. *P < 0.005.

^c^Posterior probabilities value of each codon site were showed in parentheses.

## References

[b1] Hyde K., Swe A. & Zhang K.-Q. Nematode-Trapping Fungi. In Nematode-Trapping Fungi 1–12 (Springer, 2014).

[b2] Yu Z., Mo M., Zhang Y. & Zhang K.-Q. Taxonomy of Nematode-Trapping Fungi from *Orbiliaceae*, Ascomycota. In Nematode-Trapping Fungi 41–210 (Springer, 2014).

[b3] Nordbring-Hertz B., Jansson H.-B. & Tunlid A. Nematophagous Fungi. Els 1–11 (2006).

[b4] Siddiqui Z. A. & Mahmood I. Biological control of plant parasitic nematodes by fungi: a review. Bioresource Technol 58, 229–239 (1996).

[b5] Grnvold J. *et al.* Biological control of nematode parasites in cattle with nematode-trapping fungi: a survey of Danish studies. Vet Parasitol 48, 311–325 (1993). 8346645 10.1016/0304-4017(93)90165-j

[b6] Moosavi M. R. & Zare R. Fungi as biological control agents of plant-parasitic nematodes. In Plant Defence: Biological Control (eds. M. M. J. & G. R. K.) 67–107 (Springer, 2012).

[b7] Zhang K.-Q. & Hyde K. D. Nematode-trapping Fungi. (Springer Science & Business, 2014).

[b8] Li J. *et al.* Molecular mechanisms of nematode-nematophagous microbe interactions: basis for biological control of plant-parasitic nematodes. Annu Rev Phytopathol 53, 67–95 (2015). 25938277 10.1146/annurev-phyto-080614-120336

[b9] Chakrabarti A. & Shivaprakash M. Microbiology of systemic fungal infections. J Postgrad Med 51, 16 (2005). 16519250

[b10] Wang L. & Lin X. Morphogenesis in fungal pathogenicity: shape, size, and surface. PLoS pathog 8, e1003027 (2012). 23236274 10.1371/journal.ppat.1003027 PMC3516537

[b11] Yang J. *et al.* Genomic and proteomic analyses of the fungus *Arthrobotrys oligospora* provide insights into nematode-trap formation. PLoS Pathog 7, e1002179 (2011). 21909256 10.1371/journal.ppat.1002179 PMC3164635

[b12] Tunlid A., Johansson T. & Nordbring-Hertz B. Surface polymers of the nematode-trapping fungus *Arthrobotrys oligospora*. Microbiology 137, 1231 (1991). 10.1099/00221287-137-6-1231 1919501

[b13] Heintz C. E. & Pramer D. Ultrastructure of nematode-trapping fungi. J Bacteriol 110, 1163–1170 (1972). 4113122 10.1128/jb.110.3.1163-1170.1972 PMC247540

[b14] Barron G. The nematode-destroying fungi. In Topics in Mycology 140 (Canadian Biological Publications Ltd, Guelph, Ont., Canada Guelph, Ontario, 1977).

[b15] Chen T. H., Hsu C. S., Tsai P. J., Ho Y. F. & Lin N. S. Heterotrimeric G-protein and signal transduction in the nematode-trapping fungus *Arthrobotrys dactyloides*. Planta 212, 858–863 (2001). 11346962 10.1007/s004250000451

[b16] Liu K., Tian J., Xiang M. & Liu X. How carnivorous fungi use three-celled constricting rings to trap nematodes. Protein Cell 3, 325–328 (2012). 22528749 10.1007/s13238-012-2031-8 PMC4875469

[b17] Pfister D. *Orbilia fimicola*, a nematophagous discomycete and its *Arthrobotrys* anamorph. Mycologia 86, 451–453 (1994).

[b18] Pfister D. & Liftik M. Two *Arthrobotrys* anamorphs from *Orbilia auricolor*. Mycologia 87, 684–688 (1995).

[b19] Scholler M., Hagedorn G. & Rubner A. A reevaluation of predatory orbiliaceous fungi. II. A new generic concept. Sydowia 51, 89–113 (1999).

[b20] Ahrén D. & Tunlid A. Evolution of parasitism in nematode-trapping fungi. J Nematol 35, 194–197 (2003). 19265994 PMC2620624

[b21] Ahrén D., Ursing B. M. & Tunlid A. Phylogeny of nematode-trapping fungi based on 18S rDNA sequences. FEMS Microbiol Lett 158, 179–184 (1998). 9465391 10.1111/j.1574-6968.1998.tb12817.x

[b22] Talavera G. & Castresana J. Improvement of phylogenies after removing divergent and ambiguously aligned blocks from protein sequence alignments. Syst Biol 56, 564–577 (2007). 17654362 10.1080/10635150701472164

[b23] Chen J., Xu L. L., Liu B. & Liu X. Z. Taxonomy of *Dactylella* complex and Vermispora. II. The genus *Dactylella*. Fungal Divers 26, 127–142 (2007).

[b24] Chen J., Xu L. L., Liu B. & Liu X. Z. Taxonomy of *Dactylella* complex and Vermispora. I. Generic concepts based on morphology and ITS sequences data. Fungal Divers 26, 73–83 (2007).

[b25] Li Y. *et al.* Phylogenetics and evolution of nematode-trapping fungi (*Orbiliales*) estimated from nuclear and protein coding genes. Mycologia 97, 1034–1046 (2005). 16596955 10.3852/mycologia.97.5.1034

[b26] Yang E. *et al.* Origin and evolution of carnivorism in the Ascomycota (fungi). P Natl Acad Sci, USA 109, 10960–10965 (2012). 10.1073/pnas.1120915109 PMC3390824 22715289

[b27] Yang Y., Yang E., An Z. & Liu X. Evolution of nematode-trapping cells of predatory fungi of the *Orbiliaceae* based on evidence from rRNA-encoding DNA and multiprotein sequences. *P Natl Acad Sci, USA* 104, 8379–8384 (2007). 10.1073/pnas.0702770104 PMC1895958 17494736

[b28] Liang L. *et al.* Proteomic and transcriptional analyses of *Arthrobotrys oligospora* cell wall related proteins reveal complexity of fungal virulence against nematodes. Appl Microbiol Biotechnol 97, 8683–8692 (2013). 23948728 10.1007/s00253-013-5178-1

[b29] Wang C. St Leger, R. The MAD1 adhesin of *Metarhizium anisopliae* links adhesion with blastospore production and virulence to insects, and the MAD2 adhesin enables attachment to plants. Eukaryot Cell 6, 808–816 (2007). 17337634 10.1128/EC.00409-06 PMC1899246

[b30] Liang L. *et al.* A proposed adhesin *AoMad1* helps nematode-trapping fungus *Arthrobotrys oligospora* recognizing host signals for life-style switching. Fungal Genet Biol 172–181 (2015). 25724687 10.1016/j.fgb.2015.02.012

[b31] Liu K. *et al.* *Drechslerella stenobrocha* genome illustrates the mechanism of constricting rings and the origin of nematode predation in fungi. BMC Genomics 15, 114 (2014). 24507587 10.1186/1471-2164-15-114 PMC3924618

[b32] Meerupati T. *et al.* Genomic mechanisms accounting for the adaptation to parasitism in nematode-trapping fungi. PLoS Genet 9, e1003909 (2013). 24244185 10.1371/journal.pgen.1003909 PMC3828140

[b33] White T. J., Bruns T., Lee S. & Taylor J. Amplification and direct sequencing of fungal ribosomal RNA genes for phylogenetics. PCR protocols: a guide to methods and applications 18, 315–322 (1990).

[b34] Zdobnov E. M. & Apweiler R. InterProScan - an integration platform for the signature-recognition methods in InterPro. Bioinformatics 17, 847–848 (2001). 11590104 10.1093/bioinformatics/17.9.847

[b35] Edgar R. MUSCLE: multiple sequence alignment with high accuracy and high throughput. Nucleic Acids Res 32, 1792–1797 (2004). 15034147 10.1093/nar/gkh340 PMC390337

[b36] Castresana J. Selection of conserved blocks from multiple alignments for their use in phylogenetic analysis. Mol Biol Evol 17, 540–552 (2000). 10742046 10.1093/oxfordjournals.molbev.a026334

[b37] Tamura K., Stecher G., Peterson D., Filipski A. & Kumar S. MEGA6: molecular evolutionary genetics analysis version 6.0. Mol Biol Evol 30, 2725–2729 (2013). 24132122 10.1093/molbev/mst197 PMC3840312

[b38] Ronquist F. & Huelsenbeck J. MrBayes 3: Bayesian phylogenetic inference under mixed models. Bioinformatics 19, 1572–1574 (2003). 12912839 10.1093/bioinformatics/btg180

[b39] Guindon S. & Gascuel O. A simple, fast, and accurate algorithm to estimate large phylogenies by maximum likelihood. Syst Biol 52, 696–704 (2003). 14530136 10.1080/10635150390235520

[b40] Posada D. & Crandall K. Modeltest: testing the model of DNA substitution. Bioinformatics 14, 817–818 (1998). 9918953 10.1093/bioinformatics/14.9.817

[b41] Felsenstein J. & Kishino H. Is there something wrong with the bootstrap on phylogenies? A reply to Hillis and Bull. Syst Biol 42, 193–200 (1993).

[b42] Hurst L. D. The Ka/Ks ratio: diagnosing the form of sequence evolution. Trends Genet 18, 486–487 (2002). 12175810 10.1016/s0168-9525(02)02722-1

[b43] Bielawski J. & Yang Z. Maximum likelihood methods for detecting adaptive evolution after gene duplication. J Struct Funct Genom 3, 201–212 (2003). 12836699

[b44] Bielawski J. P. & Yang Z. A maximum likelihood method for detecting functional divergence at individual codon sites, with application to gene family evolution. J Mol Evol 59, 121–132 (2004). 15383915 10.1007/s00239-004-2597-8

[b45] Yang Z. PAML 4: phylogenetic analysis by maximum likelihood. Mol Biol Evol 24, 1586–1591 (2007). 17483113 10.1093/molbev/msm088

[b46] Lewis P. O., Holder M. T. & Holsinger K. E. Polytomies and Bayesian phylogenetic inference. Syst Biol 54, 241–253 (2005). 16012095 10.1080/10635150590924208

[b47] Yang Z. Likelihood ratio tests for detecting positive selection and application to primate lysozyme evolution. Mol Biol Evol 15, 568–573 (1998). 9580986 10.1093/oxfordjournals.molbev.a025957

[b48] Yang Z., Wong W. S. W. & Nielsen R. Bayes empirical Bayes inference of amino acid sites under positive selection. Mol Biol Evol 22, 1107–1118 (2005). 15689528 10.1093/molbev/msi097

[b49] Zhang J., Nielsen R. & Yang Z. Evaluation of an improved branch-site likelihood method for detecting positive selection at the molecular level. Mol Biol Evol 22, 2472–2479 (2005). 16107592 10.1093/molbev/msi237

[b50] Nielsen R. & Yang Z. Likelihood models for detecting positively selected amino acid sites and applications to the HIV-1 envelope gene. Genetics 148, 929–936 (1998). 9539414 10.1093/genetics/148.3.929 PMC1460041

[b51] Bonferroni C. Il calcolo delle assicurazioni su gruppi di teste. In Studi in Onore del Professore Salvatore Ortu Carboni 13–60(Rome,1935).

[b52] Bonferroni C. E. Teoria statistica delle classi e calcolo delle probabilità. In Pubblicazioni del R Istituto Superiore di Scienze Economiche e Commerciali di Firenze 3–62 (Libreria internazionale Seeber, 1936).

[b53] Hoyer L. L. The ALS gene family of *Candida albicans*. Trends Microbiol 9, 176–180 (2001). 11286882 10.1016/s0966-842x(01)01984-9

[b54] Rauceo J. M. *et al.* Threonine-rich repeats increase fibronectin binding in the *Candida albicans* adhesin Als5p. Eukaryot Cell 5, 1664–1673 (2006). 16936142 10.1128/EC.00120-06 PMC1595330

[b55] Lo W.-S. & Dranginis A. M. The cell surface flocculin Flo11 is required for pseudohyphae formation and invasion by *Saccharomyces cerevisiae*. Mol Biol Cell 9, 161–171 (1998). 9436998 10.1091/mbc.9.1.161 PMC25236

[b56] de la Fuente J., Garcia-Garcia J. C., Blouin E. F. & Kocan K. M. Characterization of the functional domain of major surface protein 1a involved in adhesion of the rickettsia *Anaplasma marginale* to host cells. Vet Microbiol 91, 265–283 (2003). 12458174 10.1016/s0378-1135(02)00309-7

