# Supplementary material for: Phylogenic analysis of adhesion related genes Mad1 revealed a positive selection for the evolution of trapping devices of nematode-trapping fungi
Source: Sci Rep. 2016 Mar 4;6:22609. doi: 10.1038/srep22609 (PMC4778117; doi:10.1038/srep22609)

Phylogenic analysis of adhesion related genes *Mad1* revealed a positive selection for the evolution of trapping devices of nematode-trapping fungi

Juan Li*, Yue Liu, Hongyan Zhu, and Ke-Qin Zhang*

Laboratory for Conservation and Utilization of Bio-resources, and Key Laboratory for Microbial Resources of the Ministry of Education, Yunnan University, Kunming, 650091, P.R. China.

* Corresponding author: Juan Li and Ke-Qin Zhang

Tel: 86-871-65033805; Fax: +86-871-65034838.

E-mail address: juanli@ynu.edu.cn (Juan Li); kqzhang@ynu.edu.cn (Ke-Qin Zhang)

Figure S1: Amino acid sequence alignment for *Mad1* genes with MUSCLE 3.5.

```
                  ....|....| ....|....| ....|....| ....|....| ....|....| ....|....|
                      5          15         25         35         45         55
KT932011          KKCKFNIHHI KFRCDTTSAP VPATTSAPCT EYSCTATTEA VPTTEEAPCT EYSCTATTSE
KT932012          KKCKFNIHHI KFRCDTTSAP VPATTSAPCT EYSCTATTEA VPTTEEAPCT EYSCTATTSE
KT932013          KKCKFNIHHI KFRCDTTSAP VPATTSAPCT EYSCTATTEA VPTTEEAPCT EYSCTATTSE
KT932014          KKCKFNIHHI KFRCDTTSAP VPATTSAPCT EYSCTATTEA VPTTEEAPCT EYSCTATTSE
KT932015          KKCKFNIHHI KFRCDTTSAP VPATTSAPCT EYSCTATTEA VPTTEEAPCT EYSCTATTSE
KT932016          KKCKFNIHHI KFRCDTTSAP VPATTSAPCT EYSCTATTEA VPTTEEAPCT EYSCTATTSE
KT932017          KKCKFNIHHI KFRCDTTSAP VPATTSAPCT EYSCTATTEA VPTTSEGPCT EYSCTATTTE
KT932018          ---------- ---------- ---------- ---------- ----EPAPCT EYSCTATTTE
KT932019          APTTTEEGPC TEYSCTTSEA VPTTTEAPCT EYSCTATTEA VPTTDEAPCT EYSCTATTTE
KT932020          APTTTEEGPC TEYSCTTSEA VPTTTEAPCT EYSCTATTEA VPTTDEAPCT EYSCTATTTE
KT932021          --------PC TEYSCTTSEA VPTTTEAPCT EYSCTATTEA VPTTDEAPCT EYSCTATTTE
KT932022          APTTTEDVPC TEASCT--EP VPTTTDVPCT EASCSAT-EP VPTTDEVPCT EASCTATATE
KT932023          LPTTTEEAPC TEYSCTTTEA LPTTTEAPCT EYSCTATTEA LPTTEEAPCT EYSCTATTTE
KT932024          LPTTTEEAPC TEYSCTTTEA LPTTTEAPCT EYSCTATTEA LPTTEEAPCT EYSCTATTTE
XM_011123119      KKCKFNIHHI KFRCDTTSAP VPATTSAPCT EYSCTATTEP APTEEPAPCT EYSCTATTTE
KT932025          KKCKFNIHHI KFRCDTTSAP VPATTSAPCT EYSCTATSEA VPTTPDAPCT EYSCTATTSE
KT932026          KKCKFNIHHI KFRCDTTSAP VPVTTEAPCT EYSCTATTEA VPTTEEAPCT EYSCTATTTE
KT932027          -----NIHHI KFRCDTTSAP VPATTSAPCT EYSCTATSEA VPTTEEAPCT EYSCTATTSE
KT932028          KKCKFNIHHI KFRCDTTSEP VPITTSAPCT EYSCTATSEA VPTTDEAPCT EYSCTASTTE
KT932029          -ECKFNIHHI KFRCDTSSEA VPTTSSAPCT EYSCTGTSES VPTTSEAPCT EYSCT-TSSE
KT932030          ---------- -FRCDTTSEA VPETTSAPCT EYSCTGTSES VPTTSEAPCT EYSCT-TTSE
KT932031          KKCKFNIHHI KFRCDPTTEP VPQTTEAPCT DYGCPGTTEA QPTTEEAPCT DYSCTATATE
KT932032          KKCKFNVHHI KFRCDTTSAP VAETTSAPCT DYSCTATTEA VPTTSETPCT EYSCTATTSE
KT932033          KKCKFNVHHI KFRCDSQPEI PQTTSEGPCT DYSCTATSEA VPTTS-GPCT EYSCT-ATTE
XM_011114756      KKCKFNVHHI KFRCDTSQSV PPTTS-DVCT DYTCTATSEA VSTT--SVCT DYTCTASSSE
KT932034          KKCKFNVHHI KFRCDTSQSV PPTTS-DVCT DYTCTATSEA VSTT--SVCT DYTCTASSSE
KT932035          KKCKFNVHHI KFRCDTSQSV PPTTS-DVCT DYTCTATSEA VSTT--SVCT DYTCTASSSE
KT932036          KKCKFNVHHI KFRCDTSQSV PPTTS-DVCT DYTCTATSEA VSTT--SVCT DYTCTASSSE
KT932037          KKCKFNVHHI KFRCDTSQSV PPTTS-EVCT DYTCTATSEA VSTT--SVCT DYTCTASSSE
KT932038          KKCGFNVHHV KFYCDTTSAP APTTT-AACT DYTCTAAPAV VDTT--SVCT DYTCTAT--A
KT932039          ---------- ---------- ---------- ---CTAAPAV VDTT--SVCT DYTCTAT--A
KT932040          ----FNVHHV NFYCNTTSAP APTTT-AVCT DYTCTAAP-A VDTT--SVCT DYTCTAT--A
KT932041          KKCGFNVHHV NFYCNTTSPA APATT-AICT DYTCTAAPAA VDTT--SVCT DYTCTAT--A
KT932042          --CGYSIHHV NFYCGTTSAS APPTT-AVCT DYTCTATSAA VDTT--SVCT DYTCTAS--A
KT932043          ----YSIHHV NFYCGTTSAS VPPTT-AVCT DYTCTATSAA VDTT--SVCT DYTCTAS--A
KT932044          KKCGFNVHHI KFRCDTSVSV APTTS-EICT DYTCTATSEA AQTTTSSVCT DYTCTATTSE
KT932045          EECGFNVHHI KFRCDTSVSV APTTS-EICT DYTCTATSEA AQTTTSSVCT DYTCTATTSE
KT932046          EECGFNVHHI KFRCDAVTTT TTSSVCGATT SSTTTTTTTS STT-TSSVCN GYNCG-ATTS
KT932047          EECGFNVHHI KFRCDAVTTT TTSSVCGATT SSTTTTTTTS STT-TSSVCN GYNCG-ATT-
KI966443          KKCGFNVHHV KFRCDGVTTT TSSTTPPGVT TTSTTTTPST TTTTTAPECN GEDCPGTPSS
KT932048          ATTAVRVPVL LLLLLIATAT ATDVPPPPLP ATATVTDAAM ALG------- ----------
```

```
KT932049        --CGFNIHHI KFHCDAATTS TSSTVCGAGT SSSTTSTTSS STTATSALCN GYECVNSSST
KT932050        -KCKFNIHKI KFKCVDCPAT QPTDVPCPAT QPTDVPQPTD VPTTYNCPAT QPTDVPTQPT
KT932051        KKCKFNIHKI KFKCVDCPAT QPTDVPCPAT QPTDVPQPTD VPTTYDCPAT QPTDVPTQPT
KT932052        KKCKFNIHNI KFKCKGCPET QPTDVPCPET QPTGVPQPTD GPTTYGCPET QPTDVPTQPT
KT932053        ----LNIHHI KFKCSENPYV PPT-SESP-- ------TSE- ---------- --NPYVPQPT
KT932054        KKCKLNVHHI KFRCDTEPYV PPSETEEPCY GDNCNTTSEP APTTTVC--- --NGYDCSGT

                ....|....| ....|....| ....|....| ....|....| ....|....| ....|....|
                     65         75         85         95        105        115
KT932011        AVPTTEEAPC TEYSCTAEAI PTTEAPCTEY SCTPCTEYSC TAEAVPTTTP CTEYSCTASE
KT932012        AVPTTEEAPC TEYSCTAEAI PTTEAPCTEY SCTPCTEYSC TAEAVPTTTP CTEYSCTASE
KT932013        AVPTTEEAPC TEYSCTAEAI PTTEAPCTEY SCTPCTEYSC TAEAVPTTTP CTEYSCTASE
KT932014        AVPTTEEAPC TEYSCTAEAI PTTEAPCTEY SCTPCTEYSC TAEAVPTTTP CTEYSCTASE
KT932015        AVPTTEEAPC TEYSCTAEAI PTTEAPCTEY SCTPCTEYSC TAEAVPTTTP CTEYSCTASE
KT932016        AVPTTEEAPC TEYSCTAEAI PTTEAPCTEY SCTPCTEYSC TAEAIPTTTP CTEYSCTASE
KT932017        AVPTTDEAPC TEYSCTAEAV PTTEAPCTEY SCTPCTEYSC TAEAVPTTTP CTEYSCTATQ
KT932018        PAPTTEEGPC TEYSCTAEAV PTTEAPCTEY SCTPCTEYSC TAEAVPTTTP CTEYSCTATD
KT932019        PAPTTDEAPC TEYSCTAEAV PTTEAPCTEY SCTPCTEYSC TAEAVPTTTP CTEYSCTATE
KT932020        PAPTTDEAPC TEYSCTAEAV PTTEAPCTEY SCTPCTEYSC TAEAVPTTTP CTEYSCTATE
KT932021        PAPTTDEAPC TEYSCTAEAV PTTEAPCTEY SCTPCTEYSC TAEAVPTTTP CTEYSCTATD
KT932022        AVPTTDEAPC TEYSCTAEAV PTTEAPCTEA SCSPCTEASC TAEPAPTTTP CTEYSCTATE
KT932023        ALPTTEEAPC TEYSCTAEAL PTTEAPCTEY SCTPCTEYSC TAEAAPTTTP CTEYSCTATE
KT932024        ALPTTEEAPC TEYSCTAEAL PTTEAPCTEY SCTPCTEYSC TAEAAPTTTP CTEYSCTATE
XM_011123119    PAPTTEPAPC TEYSCTAEPA PTEPAPCTEY SCTPCTEYSC TAEPAP-TEP CTEYSCTASE
KT932025        AVPTTDEAPC TEYSCTAEAV PTTDAPCTEY SCTPCTEYSC TAEAVPTTTP CTEYSCTASE
KT932026        AVPTTEEAPC TEYSCTAEAV PTTEAPCTEY SCTPCTEYSC TAEAVPTTTP CTEYSCTASE
KT932027        AVPTTEEAPC TEYSCTAEAV PTTEAPCTEY SCTPCTEYSC TAEAVPTTTP CTEYSCTASE
KT932028        AVPTTGEAPC TEYSCTAEAV PTTEAPCTEY SCTPCTEYSC TAEAVPTTTP CTEYSCTASE
KT932029        AVPTSGETPC TEYSCTAEAI PTTEAPCTEY SCTPCTEYSC TGEAVPTTSP CTEYSCTGSE
KT932030        AVPTSEETPC TEYSCTAEAI PTTEAPCTEY SCTPCTEYSC TGEAVPTTS- CTEYSCTGSE
KT932031        AVPTTEEAPC TEYSCTAEAV PTTEAPCTEY SCTPCTEYSC TAEAVPTTTP CTEYSCTATE
KT932032        AAPTTSEAPC TDYSCTAEAV PTTEAPCTEY SCTPCTEYSC TAETAPSTTP CTDASCSATE
KT932033        AVPTTSAGPC TDYSCTAEAV PTTG-PCTEY SCTPCTEASC TAEAVPTTSP CTDYACSASE
XM_011114756    AVSTS---VC TDYTCTAEAV PTT--PCTEY SCTVCTDYTC TAEAGSTSSV CTEYSCTATE
KT932034        AVSTSS--VC TDYTCTAEAV PTT--PCTEY SCTVCTDYTC TAEAGSTSSV CTEYSCTGTE
KT932035        AVSTSS--VC TDYTCTAEAV PTT--PCTEY SCTVCTDYTC TAEAGSTSSV CTEYSCTATE
KT932036        AVSTSS--VC TDYTCTAEAV PTT--PCTEY SCTVCTDYTC TAEAGSTSSV CTEYSCTATE
KT932037        AASTSS--VC TDYTCTAEAV PTT--PCTEY SCTVCTDYTC TAEAVSTSSV CTEYSCTATE
KT932038        AVGATS--VC TDYTCTAAVD TTS--VCTDY TCTVCTDYTC TAAAVDTTSV CTDYTCTAS-
KT932039        AVDTTS--VC TDYTCTAAVD TTS--VCTDY TCTVCTDYTC TAAAVDTTSV CTDYTCTAS-
KT932040        AVDTTS--VC TDYTCTAAVD TTS--ACTDY TCTACTDYTC TAAAVDTTSV CTDYTCTAT-
KT932041        AVDTTS--VC TDYTCTAAVD TTS--VCTDY TCTVCTDYTC TAAAVDTTSV CTDYTCTAA-
KT932042        AVATTS--VC TDYTCTAAVA TTS--VCTDY TCTVCTDYTC TAAAVATTSV CTDYTCTA--
KT932043        AVATTS--VC TDYTCTAAVA TTS--VCTDY TCTVCTDYTC TAAAVATTSV CTDYTCTA--
```

```
KT932044       AAQTSEGP-C TEYSCTAAAQ TTTS-VCTDY SCTPCTEYSC TAVATTTSSV CNDYTC----
KT932045       AAQTSE---C TEYSCTAAAA TTTS-VCTDY SCTVCTEYSC TAVATTAS-V CTDYTCSDAA
KT932046       TTSSTTSSTT TSSVCNGSGT TTTT------ -----SGRAC YG-------- ------NTTT
KT932047       TTSSTTSSTT TSSVCNGSGT TTTT------ -----SGRAC YG-------- ------NTTT
KI966443       TTPSATPSST TTPECNGTPS TTTAPECNGE NCP-TSAPEC NGEATPTTTA CR-YADTTTT
KT932048       -NAGSSSTTT TSSVCYGG-- ---------- ----TAGYVC SG-------- ----------
KT932049       TTTTTTSSST TSSVCNGAGS STTSSVCNGY NS--SSTTVC NGSTTTTSVV CNGYNNSSTT
KT932050       DVPTPTDVPT TEP-CVPATQ PTDTEPCVPG SYD--TTEP- CVPGTQPTDV APGYDCPAQP
KT932051       DVPTPTDVPT TEP-CVPATQ PTDTEPCVPG SYD--TTEP- CVPGTQPTDV APGYDCPAQP
KT932052       DVPTPTDVPT DAPSCVPETQ PTDTTTCEPG SYG--TDVPS CVPGTQPTDV VPGYGCPEQP
KT932053       VPPTSYVPPT SENPYVPTEP T--SAPTTSD TPY--TSAP- --TEVPPTSE PY-----VPP
KT932054       SAPSTEPVPS ETEPYVPETE P--TAPCYGD SCS--TTEPT VPSEVPPVET CYGDSCSSAP

               ....|....| ....|....| ....|....| ....|....| ....|....| ....|....|
                   125        135        145        155        165        175
KT932011       EAVPTTTEEA PCTEYSCTGT SEAVPTTSEA APTP------ -ANTSVPYET PAPSTETLPP
KT932012       EAVPTTTEEA PCTEYSCTGT SEAVPTTSEA VPTPTDVYVP PANTSVPYET PAPSTETLPP
KT932013       EAVPTTTEEA PCTEYSCTGT SEAVPTTSEA VPTPTDVYVP PANTSVPYET PAPSTETLPP
KT932014       EAVPTTTEEA PCTEYSCTGT SEAVPTTSEA VPTPTDVYVP PANTSVPYET PAPSTETLPP
KT932015       EAVPTTTEEA PCTEYSCTG- ------TSEA VPTPTDVYVP PANTSVPYET PAPSTETLPP
KT932016       EAVPTTTEEA PCTEYSCTG- ------TSEA VPTPTDVYVP PANTSIPYET PAPSTETLPP
KT932017       AAVPTTTDEA PCTEYSCTAS -QAVPTT--- ---PTDVYVP PANTSIPYET LPPSTETLPP
KT932018       EAVPTTTDEA PCTEYSCTGT SEAVPTTSAD VPTPTDVYVP PANTSIPYET PSPNTETLPP
KT932019       EAVPTTTDEA PCTEYSCTGT SEAVPTTSED VPTPTDVYVP PANTSIPYET PSPNTETLPP
KT932020       EAVPTTTDEA PCTEYSCTGT SEAVPTTSED VPTPTDVYVP PANTSIPYET PSPNTETLPP
KT932021       EAVPTTTDEA PCTEYSCTGT SEAVPTTSED VPTPTDVYVP PANTSIPYET PSPNTETLPP
KT932022       EAVPTTTGET PCTEASCT-- TEAVPTTSED VPSPTDVYVP PANTSIPYET PSPSTETVPP
KT932023       EAVPTNTEEA PCTEYSCTGT SEAVPTTSED VPTPTDIYVP PLNTSIPYET PSPSTETVPP
KT932024       EAVPTNTEEA PCTEYSCTGT SEAVPTTSED VPTPTDIYVP PLNTSIPYET PSPSTETVPP
XM_011123119   EAVPTTTDEA PCTEYSCTGT SEAVPTTSDD VPTPTDIYVP PANTSIPYET PSPSTETLPP
KT932025       EAVPTTTEEA PCTEYSCTGT SEAVPTTSEA VPTPTDVYVP PANTSIPYET PAPTTESVPP
KT932026       ----TTTEEA PCTEYSCTGT SEAVPTTSED VPTPTDVYVP PANTSIPYET PAPSIESVPP
KT932027       QAVPTTTEEA PCTEYSCTGT SEAVPTTSEA VPTPTDVYVP PANTSIPYET PAPSTETVPP
KT932028       EAVPTTTDEA PCTEYSCTAS SEAVPTTSED VPTPTDVYVP PANTSVPYET PPQAPRLFLH
KT932029       EAVPTTSEET PCTEYSCTGT SEAVPTTSEA VPTPTDVYVP PVNTSIPYET PAPSTETVPP
KT932030       EAVPTTSEEA PCTEYSCTGT SEAVPSTSEA VPTPTDVYVP PVNTSIPYET PAPSTETVPP
KT932031       EAVPTTTEAA PCTEYGCPGT SEAVPTTTEE APCPVDVYVP PANTSMPYES QPPSTEYQPP
KT932032       EAVPTTSEGA PCTDYSCTAT SEAVPTTTGE APCTTGVYVP PANNSMPYET PAPSTESVPP
KT932033       EAVPTTSAG- PCTDYACSAS SAAVPTTEAV PTTSTNVYVP PTDYVPPGNT SVPSGNRELS
XM_011114756   EAAPTTS--- PCTEYSCTGS SGAVPTTSDV YIPPTDVYVP PMNTSVPYET LPPTTETLPP
KT932034       EAAPTTT--- PCTEYSCTGS SGAVPTTSDV YIPPTDVYVP PMNTSVPYET LPPTTETLPP
KT932035       EAAPTTT--- PCTEYSCTGS SGAVPTTSDV YIPPTDVYVP PMNTSVPYET LPPTTETLPP
KT932036       EAAPTTT--- PCTEYSCTGS SGAVPTTSDV YIPPTDVYVP PMNTSVPYET LPPTTETLPP
KT932037       EAAPTTS--- PCTEYSCTGS SGAVPTTSDV YIPPTDVYVP PMNTSVPYET LPPTTETLPP
```

```
KT932038       ---------- PCTDYSCTGG ------TN-V YVQPN-VYVP PMNTTAPYVQ --PSTNTLPP
KT932039       ---------- PCTDYSCTGG ------TN-V YVQPN-VYVP PMNTTAPYVQ --PSTNTLPP
KT932040       ---------- --TDTSSAG- ------IN-T YVQPN-VYVP PMNTTA-YVQ --PSTDTLPP
KT932041       ---------- PCTDYSCTGG ------TD-V YVQAN-AYVP PMNTSVPYVQ --PTTDTLPP
KT932042       ---------- PCTDYSCTGG T-----TN-V YVPVN-VYVP PMNTTAVYVQ --PSTQTLPP
KT932043       ---------- PCTDYSCTGG T-----TN-V YVPVN-VYVP PVNTTAVYVQ --PSTQTLPP
KT932044       ---------- PCTDYSCT-A T-----SS-- AAETG-VYVP PANTTVPYET --PS-ETTP-
KT932045       V--------- -CTDYSCTGA T-----TSSV YVPTD-VYVP PVNTSVPYET --PSTETTPP
KT932046       TTTTSGVGAV PTTTTTSS-- ---VCLGYGC NGGSVPTYIS PANTSAPY-- ----PPYNTG
KT932047       TTTTSGVGAV PTTTTTSS-- ---VCLGYGC NGGSVPTYIS PANTSAPY-- ----PPYNTG
KI966443       SDPADVDTTT TPAGVPS--- ---VCTDYTC TATDIP---S NATTTAQYT- ----PPVDT-
KT932048       ----YVPVYS PPAGVPD--- -------YGV PSGPYAP--- PSNGSTPY-- ------VPPS
KT932049       TTSASVAAQS TTSSVSST-- ---VCTDYTC TAGAAATSST PASVCTDYSC TAGAPAVYSS
KT932050       TQPTDIPTYV PSCEPG-S-C PATQPTDVPT DVPPTDIYVP PVNTSGPY-- VPP--VTDSP
KT932051       TQPTDVPTDV PSCEPG-S-C PATQPTDVPT YVPPTDIYVP PVNTTGPY-- VPPP-VTDSP
KT932052       TQPTDVPTDV PSCVPG-S-C PETQPTDVPT YVPTDTYYVP SVNTTAPY-- ETDNSVTDTP
KT932053       DTPYVPPTSE NYVPPTTS-D VYVPPTTTDV YVPPTDVYVP PANTSIPYET VPPS-ETLPP
KT932054       TDTYVPPT-D TYVPPT---D TYVPP--TDI YVPPTDVYVP PVNTSVPYET VPPAPVTDTP

               ....|....| ....|....| ....|....| ....|....| ....|....| ....|....|
                   185        195        205        215        225        235
KT932011       SGTDVYTT-P SVPVETGCPP VLPQCMETWT KITKCINSGD VKCLCPNPEY IKSVAECVEA
KT932012       SGTDVYTT-P SVPVETGCPP VLPQCMETWT KITKCINSGD VKCLCPNPEY IKSVAECVEA
KT932013       SGTDVYTT-P SVPVETGCPP VLPQCMETWT KITKCINSGD VKCLCPNPEY IKSVAECVEA
KT932014       SGTDVYTT-P SVPVETGCPP VLPQCMETWT KITKCINSGD VKCLCPNPEY IKSVAECVEA
KT932015       SGTDVYTT-P SVPVETGCPP VLPQCMETWT KITKCINSGD VKCLCPNPEY IKSVAECVEA
KT932016       SGTDVYTT-P SVPVETGCPP VLPQCMETWT KITKCINSGD VKCLCPNPEY IKSVAECVEA
KT932017       SGTDVYTT-P SVPVETGCPP VLPQCMETWT KITKCIDSGD VKCLCPNPEY IKSVAECVEA
KT932018       SGTDVYTT-P SVPVETGCPP VLPQCMETWT KITNCINSGD VKCLCPNPEY IKSVAECVEA
KT932019       SGTDVYTT-P SVPVETGCPP VLPQCMETWT KITNCINSGD VKCLCPNPEY IKSVAECVEA
KT932020       SGTDVYTT-P SVPVETGCPP VLPQCMETWT KITNCINSGD VKCLCPNPEY IKSVAECVEA
KT932021       SGTDVYTT-P SVPVETGCPP VLPQCMETWT KITNCINSGD VKCLCPNPEY IKSVAECVEA
KT932022       SGTDVYTT-P SVPVETGCPP VLPQCMETWT KITSCINSGD VKCLCPNPEY IKSVAECVEA
KT932023       SGTDVYTT-P SVPVETGCPP VLPQCMETWT KITQCVNSGD VKCLCPNPEY IKSVAACVEA
KT932024       SGTDVYTT-P SVPVETGCPP VLPQCMETWT KITQCVNSGD VKCLCPNPEY IKSVAACVEA
XM_011123119   SGTDVYTT-P SVPVETGCPP VLPQCMETWT KITQCVNSGD VKCLCPNPEY IKSVAECVEA
KT932025       SGTDVYTT-P SVPVETGCPP VLPQCMETWT KITQCVNSGD VKCLCPNPEY IKSVAECVEA
KT932026       TGTDVYTT-P SVPVETGCPP VLPQCMETWT KITQCVNSGD VKCLCPNPEY IKSVAECVEA
KT932027       SGTDVYTT-P SVPVETGCPP VLPQCMETWT KITKCINSGD VKCLCPNPEY IKSVAECVEA
KT932028       PGTDVYTT-P SVPVETGCPP VLPQCMETWT KITKCIDSGD VKCLCPNPEY IKSVAECVEA
KT932029       SGTDVYTT-P SVPVETGCPP VLPQCMKTWT KITQCIDSGD VKCLCPNPEY IKSVAECVEA
KT932030       SGTDVYTT-P SVPVETGCPP VLPQCMKTWT KITQCIDSGD VKCLCPNPEY IKSVAECVEA
KT932031       ATTDSYET-P PVPVETGCPP VLPQCMKTWT KITQCINSGD VKCLCPNPEY IKNVADCVEA
KT932032       SGTDVYTT-P SVPVETGCPN VLPQCMKTWT KITQCIDSGD VKCLCPNPDY INNVAACVEA
```

```
KT932033        HSAHVYTT-P SVPVETGCPP VLPQCMETWT KITKCIDSGD VKCLCPNPEY IKNVASCVEA
XM_011114756    AGTDVYTT-P SVPVETGCPP VLPQCMETWT KITQCINSGD VKCLCPNPEY IKNVAACVEA
KT932034        AGTNVYTT-P SVPVETGCPP VLPQCMETWT KITQCINSGD VKCLCPNPEY IKNVAACVEA
KT932035        AGTNVYTT-P SVPVETGCPP VLPQCMETWT KITQCINSGD VKCLCPNPEY IKNVAACVEA
KT932036        AGTNVYTT-P SVPVETGCPP VLPQCMETWT KITQCINSGD VKCLCPNPEY IKNVAACVEA
KT932037        AGTDVYTT-P SVPVETGCPP VLPQCMETWT KITQCINSGD VKCLCPNPEY IKNVAACVEA
KT932038        SGTDVYTT-P SVPVETGCPQ VLPQCMQVWT KITQCIDSGD VKCLCPNAEY IQNVASCVEA
KT932039        SGTDVYTT-P SVPVETGCPQ VLPQCMQVWT KITQCIDSGD VKCLCPNAEY IQNVASCVEA
KT932040        SGTDVYTT-P SVPVETGCPQ VLPQCMQVWT KITQCIDSGD VKCLCPNAEY IQNVASCVEA
KT932041        SGTDVYTT-P SVPVETSCPQ VLPQCMQVWT KITQCIDSGD VKCLCPNAEY IQNVASCVEA
KT932042        AGTDVYTT-P SVPVETSCPQ ILPQCMKVWT KITQCIDSGD VSCLCPNAQY IQNVASCVEA
KT932043        AGTDVYTT-P SVPVETSCPQ ILPQCMKVWT KITQCIDSGD VSCLCPNAQY IQNVASCVEA
KT932044        AGTDVYTT-P SVPVVTGCPP VLPQCMQTWT KITQCIDSGD VKCLCPNPDY IQNVAACVEA
KT932045        AGTNIYTT-P SVPVVTGCPP VLPQCMQTWT KITQCIDSGD VKCLCPNPDY IQNVASCVEA
KT932046        SAPAVTTT-P SVPIVTNCPN VLPQCMDTWT KITQCINSGD TKCLCPNADY INNVAACVEA
KT932047        SAPAVTTT-P SVPIVTNCPN VLPQCMDTWT KITQCINSGD TKCLCPNADY INNVAACVEA
KI966443        DVPAVTTT-P PVPVVTNCPP VLPQCMDTWT KITQCVNSGD IACLCPNTEY INNVAQCVEA
KT932048        SAAVPTTT-P PVPVVTNCPN VLPKCMDTWT KITKCINSGD VECLCPNTDY INNVAQCVEA
KT932049        PAASVTTT-P SVPVVTNCPS VLPQCMATWT KITQCINSGD VKCLCPNEEY INNVANCVEA
KT932050        TIITDVYPSP SVPVETGCPQ VLPQCMTAWT SITKCIDSGD VNCLCPNQDY INNVAGCVEA
KT932051        TT-TDVYPSP SIPVETGCPQ VLPKCMTVWT SITQCIDSGD VNCLCPNQEY INSVAGCVEA
KT932052        AQTTDYAP-P SVPVETGCPQ VLPKCMTAWT SITKCIDSGD VNCLCPNAEY INSVAGCVEA
KT932053        SESETAPP-- PVPVETGCPP VLPQCMATWT KITQCISAGD TKCLCPNPDY IKAVAECVEA
KT932054        VSTTDYTP-- SVPVETGCPP VLPQCMTTWT KITQCIDSGD VKCLCPNAEY IKNVAECVEA

                ....|....| ....|....| ....|....| ....|....| ....|....| ....|....|
                    245        255        265        275        285        295
KT932011        WGVDDDEVSK ALEYMQGLCA EHIPENPAIV TCVPTYVTLP PVTTGASTIT VSTTVVVPCT
KT932012        WGVDDDEVSK ALEYMQGLCA EHIPENPAIV TCVPTYVTLP PVTTGASTIT VSTTVVVPCT
KT932013        WGVDDDEVSK ALEYMQGLCA EHIPENPAIV TCVPTYVTLP PVTTGASTIT VSTTVVVPCT
KT932014        WGVDDDEVSK ALEYMQGLCA EHIPENPAIV TCVPTYVTLP PVTTGASTIT VSTTVVVPCT
KT932015        WGVDDDEVSK ALEYMQGLCA EHIPENPAIV TCVPTYVTLP PVTTGASTIT VSTTVVVPCT
KT932016        WGVDDDEVSK ALEYMQGLCA EHIPENPAIV TCVPTYVTLP PVTTGASTIT VSTTVVVPCT
KT932017        WGVDDDEISK ALEYMQGLCA EHIPENPAIV TCVPTYVTLP PVTTGASTIT VSTTVVVPCT
KT932018        WGVDDDEVSK ALEYMQGLCA EQIPENPAIV TCVPTYVTLP PVTTGASTIT VSTTVVVPCT
KT932019        WGVDDDEVSK ALEYMQGLCA EQIPENPAIV TCVPTYVTLP PVTTGASTIT VSTTVVVPCT
KT932020        WGVDDDEVSK ALEYMQGLCA EQIPENPAIV TCVPTYVTLP PVTTGASTIT VSTTVVVPCT
KT932021        WGVDDDEVSK ALEYMQGLCA EQIPENPAIV TCVPTYVTLP PVTTGASTIT VSTTVVVPCT
KT932022        WGVDDDEVSK ALEYMQGLCA EHIPENPAIV TCVPTYVTLP PVTTGASTVT ISTTVVVPCT
KT932023        WGVDDDEVSK ALEYMQGLCA EHIPENPAIV TCVPTYVTLP PVTTGASTIT VSTTVVVPCT
KT932024        WGVDDDEVSK ALEYMQGLCA EHIPENPAIV TCVPTYVTLP PVTTGASTIT VSTTVVVPCT
XM_011123119    WGVDDDEVAK ALEYMQGLCA EHIPENPAIV TCVPTYVTLP PVTTGASTVT VSTTVVVPVT
KT932025        WGVDDDEVSK ALEYMQGLCA EHIPENPAIV TCVPTYVTLP PVTTGASTIT VSTTVVVPCT
KT932026        WGVDDDEVSK ALEYMQGLCA EHIPENPAIV TCVPTYVTLP PVTTGASTIT VSTTVVVPCT
```

KT932027	WGVDDDEVSK	ALEYMQGLCA	EHIPENPAIV	TCVPTYVTLP	PVTTGASTIT	VSTTVVVPCT
KT932028	WGVDDDEVSK	ALEYMQGLCA	EHIPENPAIV	TCVPTYVTLP	PVTTGASTIT	VSTTIVVPYT
KT932029	WGVDDDEVSK	ALEYMQGLCA	EHIPENPAIV	TCVPTYVTLP	PVSSGASTIV	VSTTVVVPCT
KT932030	WGVDDDEVSK	ALEYMQGLCA	EHIPENPAIV	TCVPTYVTLP	PVSSGASTIT	VSTTVVVPCT
KT932031	WGVDDDEVAK	ALEYMQGLCA	DHIPENPAIV	TCVPSYVSLP	PASTGATTIT	VSTTIVVPCT
KT932032	WGNDDDEVAK	ALEYIQRLCA	DHIPENPAIV	TCVPTYVTIP	PATEGATTIT	VSTTVVVPCT
KT932033	WGVDDAEVAK	ALEYMQGLCA	DHIPSNPAIV	TCVPSYVTLP	PATGGVTTIT	VSTTVVVPCT
XM_011114756	WGVDDDEVAK	ALEYMQGLCA	DQIPENPAIV	TCVPTYVSLP	PASGGATTIT	VSTTVIVPCT
KT932034	WGVDDDEVAK	ALEYMQGLCA	DQIPENPAIV	TCVPTYVSLP	PASGGATTIT	VSTTVVVPCT
KT932035	WGVDDDEVAK	ALEYMQGLCA	DQIPENPAIV	TCVPTYVSLP	PASGGATTIT	VSTTVVVPCT
KT932036	WGVDDDEVAK	ALEYMQGLCA	DQIPENPAIV	TCVPTYVSLP	PASGGATTIT	VSTTVVVPCT
KT932037	WGVDDDEVAK	ALEYMQGLCA	DHIPENPAIV	TCVPTYVSLP	PAT-GATTIT	VSTTVVVPCT
KT932038	WGSDDSEVAK	ALEYMQGLCA	DHIPSNPAIV	TCVPTYVTLP	PAT-GATTIT	VSTTVVVPCT
KT932039	WGSDDSEVAK	ALEYMQGLCA	DHIPSNPAIV	TCVPTYVTLP	PAT-GATTIT	VSTTVVVPCT
KT932040	WGSDDSEVVK	ALEYMQGLCA	DHIPSNPAIV	TCVPTYVTLP	PAT-GATTIT	VSTTVVVPCT
KT932041	WGSDDSEVAK	ALEYMQGLCA	DHIPSNPAIV	TCVPTYVSLP	PAT-GATTIT	VSTTVVVPCT
KT932042	WGSDDAEVAK	ALEYMQGLCA	EHIPSNPAIV	TCVPTYVTLP	PAT-GATTIT	VSTTVVVPCT
KT932043	WGSDDAEVAK	ALEYMQGLCA	EHIPSNPAIV	TCVPTYVTLP	PAT-GATTIT	VSTTVVVPCT
KT932044	WGVDDDEVAK	ALEYMQGLCA	EHIPQNPAIV	TCVPTYVSLP	PAA-GATTIT	VSTTVVVPCT
KT932045	WGVDDDEVAT	ALEYMQGLCG	EHIPQNPAIV	TCVPTYVSLP	PAA-GATTIT	VSTTVVVPCT
KT932046	WSDDDTQIAQ	AIEFMRGLCA	EYIPTNPAIV	TAVPTYVTLP	PVT-GASTVI	VSTTITVPCT
KT932047	WSDDDTQIAQ	AIEFMRGLCA	EYIPTNPAIV	TAVPTYVTLP	PVT-GASTVI	VSTTITVPCT
KI966443	WSGDDAEVGR	ALEYMQGLCA	DYIPGNPAIV	TAVPSYVTLP	PVS-GASTVV	VSTTITVPYT
KT932048	WSSDDSEVIK	ALQYMQGLCA	QYIPSNPAIV	TAVPSYVTVP	PVSSGATTIT	VSTVVTVPCT
KT932049	WSNDDAEIER	ALQYMRGLCA	QYIPGNPAIV	TAVPSYVTVA	PVSAGATTVV	VTQTLTVPCT
KT932050	YGVDDDEVAK	ALEYLQGLCA	EKIPENPAII	TCVPTYVTLP	PATSGASTIT	VSTTVVVPCT
KT932051	YGVDDDEVAK	ALEYLQGLCA	EKIPENPAIV	TCVPTYVTLP	PATSGASTIT	VSTTVVVPCT
KT932052	YGVDDDEVAK	ALEYLQGLCA	EKIPENPAIV	TCVPTYVTLP	PVTSGASTVT	ISTTVVVPCT
KT932053	WGVDDDEVSK	ALEYMQGLCA	EHIPENPAIV	TCVPTYVPLP	PVTSGASTIT	ISTTVVVPCT
KT932054	WGVDDDEVAK	ALEYMQGLCA	EQIPENPAIV	TCIPTYVTLP	PATSGATTVT	ISTTVVVPCT

	....|....|	....|....|	....|....|	....|....|	....|....|	....|....|
	305	315	325	335	345	355
KT932011	TAPPEETTQP	GYVPSYTTET	VVKTVTVCPV	KLVTTEPSKP	VLVPGTITAP	PYVPAPATYP
KT932012	TAPPEETTQP	GYVPSYTTET	VVKTVTVCPV	KLVTTEPSKP	VLVPGTITAP	PYVPAPATYP
KT932013	TAPPEETTQP	GYVPSYTTET	VVKTVTVCPV	KLVTTEPSKP	VLVPGTITAP	PYVPAPATYP
KT932014	TAPPEETTQP	GYVPSYTTET	VVKTVTVCPV	KLVTTEPSKP	VLVPGTITAP	PYVPAPATYP
KT932015	TAPPEETTQP	GYVPSYTTET	VVKTVTVCPV	KLVTTEPSKP	VLVPGTITAP	PYVPAPATYP
KT932016	TAPPEETTQP	GYVPSYTTET	VVKTVTVCPV	KLVTTEPSKP	VLVPGTITAP	PYVPAPATVP
KT932017	TAPPEETTQP	GYVPSYTTET	VVKTVTVCPV	KLVTTEPSKP	VLVPGTITAP	PYVPAPATVP
KT932018	TAPPEETAQP	GYVPSYTTET	VVKTVTVCPV	KLVTTEPSKP	VLVPGTITAP	PYVPVPATYP
KT932019	TAPPEETAQP	GYVPSYTTET	VVKTVTVCPV	KLVTTEPSKP	VLVPGTITAP	PYVPVPATYP
KT932020	TAPPEETAQP	GYVPSYTTET	VVKTVTVCPV	KLVTTEPSKP	VLVPGTITAP	PYVPVPATYP
KT932021	TAPPEETAQP	GYVPSYTTET	VVKTVTVCPV	KLVTTEPSKP	VLVPGTITAP	PYVPVPATYP

KT932022 TAPPEETNKP GYVPSYTTET VVKTVTVCPV KLVTTEPSKP VLVPGTITAP PYVPAPATVP
KT932023 TASPEETTKP GYVPSYTTET IVKTVTVCPV KLVTTEPSKP VLVPGTITAP AYIPAPATIP
KT932024 TASPEETTKP GYVPSYTTET IVKTVTVCPV KLVTTEPSKP VLVPGTITAP AYIPAPATIP
XM_011123119 TASPEETNKP GYVPVFTTET VIRTVTVCPV KLVTTEPSKP VLVPGTITAP PYVPAPATIP
KT932025 TAPPEETNQP GYVPSYTTET VVKTVTVCPV KLVTTEPSKP VLVPGTITAP PYIPAPATIP
KT932026 TAPPEETTQP GYVPSYTTET VVKTVTVCPV KLVTTEPSKP VLVPGTITAP PYVPAPATVP
KT932027 TASPEETNKP DYVPSYTTET VVKTVTVCPV KLVTTEPSKP VLVPGTITAP PYVPAPATVP
KT932028 TASPEETEKP DYVPSYTTQT VIKTVTVCPV KLVTTEPSKP VLVPGTITAP PYVPAPATYP
KT932029 TAPPEETNQP NYVPSYTTET VVKTVTVCPV KLVTTEPSKP VLVPGTITAP PYVPAPATVP
KT932030 TAPPEETNQP NYVPSYTTET VVKTVTVCPV KLVTTEPSKP VLVPGTITAP PYIPAPATVP
KT932031 TAPPEETNMP GYTPSMVTEV VATTVTVCPV KLVTTEPSKP VLVPGTITAP PYVPS-ETAP
KT932032 TASPEETNQP NYVPSYTTQI VATTVTVCPV KLVTTEPSKP VLVPGTITAP PYVPAPSTMP
KT932033 NIPAQGTTAP GYVPSYTTQT VATTVTVCPV KLVTTEPAKP VLVPGTITAP PYVPAPTPVP
XM_011114756 TEPASVTEKP GYVPSYITSV VATTVTVCPV KLVTTEPSKP VLVPGTITAP PYVPAPSTMP
KT932034 TEPASVTEKP GYVPSYITEV VATTVTVCPV KLVTTEPSKP VLVPGTITAP PYVPAPSTMP
KT932035 TEPASVTEKP GYVPSYITEV VATTVTVCPV KLVTTEPSKP VLVPGTITAP PYVPAPSTMP
KT932036 TEPASVTEKP GYVPSYITEV VATTVTVCPV KLVTTEPSKP VLVPGTITAP PYVPAPSTMP
KT932037 TAPASVTNQP GYVPSYTTQI VATTVTVCPV KLVTTEPSKP VLVPGSVTAP PYVPAPSTMP
KT932038 NVAASVTTAA GYTPSYTTQV VATTVTVCPV KLVTTVPNKP VLVPATVTAP PYVPAPSSVP
KT932039 NVAASVTTAA GYTPSYTTQV VATTVTVCPV KLVTTVPNKP VLVPATVTAP PYVPAPSSVP
KT932040 NVAASITTAA GYVPSYTTQV VATTVTVCPV KLITTVPNKP VLVPATVTAP PYVPAPSSVP
KT932041 NVAADVTTAA GYIPSYTTQV IATTVTVCPV KLITTVPNKP VLVPATVTAP PYVPAPSSMP
KT932042 GVAASITTAA GYVPSYTTQV VATTVTVCPV KLVTTAPNKP VLVPATVTAP PYVPAPSSMP
KT932043 GVAASITTAA GYVPSYTTQV VATTVTVCPV KLVTTAPNKP VLVPATVTAP PYVPAPSSMP
KT932044 NVPAAVTTAA GYVPSYTTQI VATTVTVCPV KLVTTEPSKP VLVPGSVTAP PYVPAPSTMP
KT932045 NVPASVTSAA GYVPSYTTQI VATTVTVCPV KLVTTEPSKP VLVPGSVTAP PYVPAPSTMP
KT932046 ---AQTT--- ----SYSTQV IATTVTVCPV KLVTTEPAKP VLVPGTVTAP PYVPAPSTMP
KT932047 ---AQTT--- ----SYSTQV IATTVTVCPV KLVTTEPAKP VLVPGTVTAP PYVPAPSTMP
KI966443 APPGTETPG- -AVPVYSTQV IATTITVCPV KLVATDPAKP VLVPGTVTAP AYVPAP-TVP
KT932048 NQPATGAG-- YAAPSYTTQI IATTVTVCPV KLVTTASAVP VLVPGTVSAP PYVPAP-TVP
KT932049 NSAAVTTAAV PVAPSYTTQV IATTVTVCPV KLITTASAQP VLVPGTVTAP PYVPAPPTMP
KT932050 SCAPEETTAP GYQPVYYVTET IATTVTVCPV KLVTTEPSKP VLVPGTVTAP PYVP-PSSVP
KT932051 SCAPEQTTAP GYEPVYYVTET IATTVTVCPV KLVTTEPSKP VLVPGTVTAP PYVP-PSSVP
KT932052 TCAPAETTAP GYQPVYITET IATTVTVCPV KLVTTEPSKP VLVPGTVTAP PYVP-ASSVP
KT932053 TAPPEETEKP GYTTSYITET VATTVTVCPV KLVTTEPGKP VLVPGTVTAP PYVPAPVVLP
KT932054 TAPPEETTQP GYTPSYTTEV VATTVTVCPV KLVTTEPNKP VLVPGTVTAP PYVPTASSMP

....|....| ....|....| ....|....| ....|....| ....|....| ....|....|
365 375 385 395 405 415

KT932011 ATVPAEATTP -VAYVPSTLV TAVPTAPVNS TTPNPPIATG AASSFKAFST VMLAGVIGLT
KT932012 ATVPAEATTP -VEYVPSTLV TAVPTAPVNG TTPNPPIATG AASSFKAFST VMLAGVIGLT
KT932013 ATVPAEATTP -VEYVPSTLV TAVPTAPVNG TTPNPPIATG AASSFKAFST VMLAGVIGLT
KT932014 ATVPAEATTP PVEYVPSTLV TAVPTAPVNG TTPNPPIATG AASSFKAFST VMLAGVIGLT
KT932015 ATVPAEATTP PVEYVPSTLV TAVPTAPVNG TTPNPPIATG AASSFKAFST VMLAGVIGLT

KT932016	ATVPAEATTP	PVAYVPSTLV	TAVPTAPVNN	TSPNPPVATG	AASSFKAFST	VMLAGVIGLT
KT932017	ATVPAQATPP	-VAYVPSTLV	TAVPTAPANT	TGPNPPIATG	AASSFKAFST	VMLAGVIGLT
KT932018	ATVPAEATTP	PVDYVPSTMV	TAYPTAPVNS	TNPNPPIATG	AASSVKAFST	VILAGVIGLT
KT932019	ATVPAEATTP	PVDYVPSTMV	TAYPTAPVNS	TNPNPPIATG	AASSVKAFST	VILAGVIGLT
KT932020	ATVPAEATTP	PVDYVPSTMV	TAYPTAPVNS	TNPNPPIATG	AASSVKAFST	VILAGVIGLT
KT932021	ATVPAEATTP	PVDYVPSTMV	TAYPTAPVNS	TNPNPPIATG	AASSVKAFST	VILAGVIGLT
KT932022	ATIPAEATTP	PVEYAPSTMV	TAYPTVPANT	TAPRPPIATG	AASSFRAFST	VMLAGVIGLT
KT932023	ATVPAEATTP	PVEYVPSTMV	TAYPTVPANT	TVPNPPIATG	AASSFKAFST	VMLA-VIGLT
KT932024	ATVPAEATTP	PVEYVPSTMV	TAYPTVPANT	TVPNPPIATG	AASSFKAFST	VMLA-VIGLT
XM_011123119	ATVPAEATTP	PVEYAPSTLM	TAYPTVPVNN	TTPNPPIATG	AASSFKAFST	VMLAGVIGLT
KT932025	ATVPAEATTP	PVEYAPSTLM	TAYPTVPANN	TTPNPPIATG	AASSFKAFST	VMFAGVIGLT
KT932026	ATIPAEATTP	PVEYVPSTMV	TAYPTVPVNE	TTPNPPIATG	AASSFKAFST	VMLAGVIGLT
KT932027	ATVPAEATTP	PVEYVPSTLV	TAVPTVPVNG	TTPSPPIATG	AASSFKAFNT	VMLAGVIGLT
KT932028	ATVPAEATPP	PAEYVPSTMV	TAVPTAPVNG	TTPNPPIATG	AGSSFKAFST	VMLAGVIGLT
KT932029	ATVPAEATTP	PVAYVPSTLV	TSTPTPPYNG	TTPNPPVATG	AASPFKAFST	VMLAGVVGLT
KT932030	ATIPAEATTP	PAAYVPSTLV	TATPTPPYNG	TNPNPPVATG	AASSFKTFST	VMLAGVIGLT
KT932031	ASVPAEYTPT	VPVYEPTTMV	TAVPTNPVNS	TNPYPPVATG	AASSMKSFTG	LLAAGIVGVV
KT932032	ATVPAEYTT-	-PAYIPSTLA	TAYPTGPVNS	TNPSPPVATG	AASSLKVFSS	VLVAGVIGVA
KT932033	ATVPAGYTT-	V-AYVPTTMA	TAYPTGGVNN	TNPYPPVATG	AASSIKGFGT	LMVAGVLGAA
XM_011114756	ATIPAQYTT-	-PVYVPTTMA	TAYPTG--NG	TNPNPPVATG	AASSIKAFSS	FMAVGIIGVV
KT932034	ATMPAQYTT-	-PVYVPTTMA	TAYPTGPGNG	TTPNPPIATG	AASSIKAFSS	LMAVGIIGVV
KT932035	ATMPAQYTT-	-PVYVPTTMA	TAYPTGPGNG	TTPNPPIATG	AASSIKAFSS	LMAVGIIGVV
KT932036	ATMPAQYTT-	-PVYVPTTMA	TAYPTGPGNG	TTPNPPIATG	AASSIKAFSS	LMAVGIIGVV
KT932037	ATIPAEYTT-	-PVYVPTTMA	TAYPTGPGNG	TTPNPPVATG	AASSIKAFSS	LMAAGIIGVV
KT932038	ATVPVQYTTP	VPTYVPTTMA	TAYPTGSGNG	TTPYPPVATG	AGSSVKVFTS	LMA-GFIGVA
KT932039	ATVPVQYTTP	VPTYVPTTMA	TAYPTGSGNG	TTPYPPVATG	AGSSVKVFTS	LMA-GFIGVA
KT932040	VNVPVQYTTP	VPTYVPTTMA	TAYPTGSGNG	TTPYPPVATG	AGSSVKVFTS	LMA-GFLGVA
KT932041	ATVPVQYTTP	VPTYVPTTMA	TAYPTDSGNG	TTPYPPVATG	AGSSVKVFTS	LMA-GFIGVA
KT932042	ATVPVQYTT-	VPTYVPTTMA	TAYPTNPGNS	TTPYVPVATG	AGSSVKVFTS	LMAAGVIGVV
KT932043	ATVPVQYTT-	VPTYVPTTMA	TAYPTNPGNS	TTPYVPVATG	AGSSVKVFTS	LMAAGVIGVV
KT932044	ATVPAQYTT-	-PVYVPTTMA	TAYPTGPGNT	TNPNPPIATG	AASSIKVFSS	LMAAGIIGAV
KT932045	ATVPAQYTT-	-PVYAPTTLA	TAYPTGPGNT	TNPNPPVATG	AASSIKVFSS	LMAAGIIGAV
KT932046	ATVPV-Y---	QPTVAPSTLV	T-YPAAPN-V	TNPNPPVATG	SASSIKAFGS	LALAGVISVV
KT932047	ATVPV-Y---	QPTVAPSTLV	T-YPAAPN-V	TNPNPPVATG	SASSIKAFGS	LALAGVISVV
KI966443	ATTPVDN---	YTVYVPSTLV	TAIPTYVN-V	TTPNPPVATG	SASSVKAFGS	IALAGVISIV
KT932048	ATQPAVQ---	PTAYVPTTMV	SAYPTPGNNG	TAPPPPVATG	AASSVKAFSS	LALAG--ALV
KT932049	ATAPVA----	VPTYVPSTLA	TAYPTAPN-S	TTPNPPVATG	AASSIQALSS	LALAGVIGIL
KT932050	ATIPAQ-PT-	--EYFPTTMA	TLYPTAP-NT	TIDTPPVATG	AASSLKAMTG	LMGAGMVGVV
KT932051	ATVPAQ-PT-	--EYVPTTMA	TFYPTAP-NT	TINNPPVATG	AASSVKVFTG	FMGAGMVGVI
KT932052	ATIPAQ-PT-	--EYLPTTMA	TLYPTAP-NT	TVNSPPVATG	AASSLKAVSS	LMAVGIVGVV
KT932053	TTVPAQ-PTP	--VYAPTTME	TLYPTAP-NT	TNPLPPVATG	AASSLKVFSS	VMAAGIVGVA
KT932054	STAPVDYPTS	--VYAPTTMA	TVYPTAP-NA	TTSAPPIATG	AAS-LNTFSS	LMAAGIVGAV

....

KT932011	ALVM
KT932012	ALIM
KT932013	ALIM
KT932014	ALIM
KT932015	ALIM
KT932016	ALIM
KT932017	ALIM
KT932018	ALIM
KT932019	ALIM
KT932020	ALIM
KT932021	ALIM
KT932022	ALIM
KT932023	ALIM
KT932024	ALIM
XM_011123119	ALIM
KT932025	ALIM
KT932026	ALIM
KT932027	ALIM
KT932028	ALIM
KT932029	ALIM
KT932030	ALIM
KT932031	ALIM
KT932032	ALIM
KT932033	ALIL
XM_011114756	ALIM
KT932034	ALIM
KT932035	ALIM
KT932036	ALIM
KT932037	ALIM
KT932038	ALIV
KT932039	ALIV
KT932040	TLIM
KT932041	ALIM
KT932042	ALIM
KT932043	ALIM
KT932044	ALIM
KT932045	ALIM
KT932046	ALIM
KT932047	ALIM
KI966443	ALIL
KT932048	ALIM
KT932049	ALIM
KT932050	ALIM

KT932051	ALIM
KT932052	ALIM
KT932053	ALIM
KT932054	ALIM

Figure S2: Alignment of ITS fragments with MUSCLE 3.5.

```
                ....|....| ....|....| ....|....| ....|....| ....|....| ....|....|
                       5         15         25         35         45         55
KT932055        CATTTGTGAA CCAAAACAA- ACCTTTCGCT TCGGC-AGCA G--------- --GGCCCTAA
KT932056        CCTTTGTGAA CCAAAA-AA- CCTTTTCGCT TCGGC-AGCT G-------GG CTCTCCCCTA
KT932057        CCTTTGTGAA CCAAAA-AA- CCTTTTCGCT TCGGC-AGCT G--------- --GGCCTCA-
KT932058        CCTTTGTGAA CCAAAA-AA- CCTTTTCGCT TCGGC-AGCT G--------- --GGCCTCA-
KT932059        CCTCTGTGAA CCAAAA-AA- -ACATTCGCT TCGGC-GGCA G-------CC TCGGTCGGAG
KT932060        CCTCTGTGAA CCAAAA-AA- -ACATTCGCT TCGGC-GGCA G-------CC TCGGTCGGAG
KT932061        CCTTTGTGAA CCAAAA-AA- -CCTTTCGCT TCGGC-AGCA G--------T CGGGTCAGGA
KT932062        CCTTTGTGAA CCAAAA-AA- -CCTTTCGCT TCGGC-AGCT G--------- ----------
KT932063        CCTCTGTGAA CCAAAA-AA- -CCTTTCGCT TCGGC-AGCA G--------C TCGGTTGGCA
KT932064        CCTTTGTGAA CCAAAA-AA- -CCTTACGCT TCGGC-AGCA G------GTC TAGGTCAGAA
KT932065        CCTTTGTGAA CCAAAA-AA- -CCTTTCGCT TCGGC-AGCT G-------GG CCAACCCTGA
KT932066        CCTTTGTGAA CCAAAA-AA- -CCTTTCGCT TCGGC-AGCT G--------- ----------
KT932067        CCTTTGTGAA CCAAAA-AA- -CCTTTCGCT TCGGC-AGCG G--------- ----------
KT932068        CCTTTGTGAA CCAAAA-AA- CACTTTCGCT TCGGC-AGCT G--------- --GGCTTTAA
KT932069        CCTTTGTGAA CCAAAA-AA- CCTTTTCGCT TCGGC-AGCT G--------- --GGCCTGA-
KT932070        CCTTTGTGAA CCAAAA-AA- CCTTTTCGCT TCGGC-AGCT G--------- --GGCCTGA-
KT932071        CCTTTGTGAA CCAAAA-AA- CCTTTTCGCT TCGGTTAGCG GGGAGCGGTT GCGGGTGAAA
KT932072        CCTTTGTGAA CCAAAA-AA- -CCTTTCGCT TCGGC-AGCT G--------- --GGTCCTAA
KT932073        CCTTTGTGAA CCAAAA---- TCTTTACGCT TCGGC-AGCA G--------- ---GCCTTA-
KT932074        CCTTTGTGAA CCAAAA-AA- CACTTTCGCT TCGGT-AGCT G--------- --GGCCCTAA
KT932075        CCTTTGTGAA CCAAAACCA- --TTTTCGCT TCGGC-AGCT G--------- --GGGCTC--
KT932076        CCTTTGTGAA CCAAAA-AA- -CCTTTCGCT TCGGC-AGCT G--------- ----------
KT932077        CCTTTGTGAA CCAAAA-AA- CCTTTTCGCT TCGGC-AGCT G--------- --GGCCTCA-
KT932078        CCTTTGTGAA CCAAAA-AA- -CCTTTCGCT TCGGC-AGCA G--------C TCGGTTGGGA
KT932079        CCTTTGTGAA CCAAAC---C TTTCTTCGCT TCGGC-AGCA A--------- -------TGG
KT932080        CCTTTGTGAA CCAAAACCA- --TTTTCGCT TCGGC-AGCT G--------- --GGGCTC--
KT932081        CCTTTGTGAA CCAAAA-AAA CCTTTTCGCT TCGGC-AGCT G--------- --GGCCTGA-
KT932082        CCTTTGTGAA CCAAAA-AA- -CCTTTCGCT TCGGC-AGCA G--------C CTGGTTGGAA
KT932083        CCTTTGTGAA CCAAAACAA- -CCTTACGCT TCGGC-AGCT G-------GC TCTGAGCAGA
KT932084        CCTTTGTGAA CCAAAA-AA- -CCATTCGCT TCGGC-AGCA G--------C CAGGTCAGAA
KT932085        CTTTTGTGAA CCAAAA-AAA CCATTTCGCT TCGGC-AGCT GGGTCTTCGT CCGGTCTTGA
KT932086        CCTTTGTGAA CCAAAA-AA- CCTTTTCGCT TCGGC-AGCT G--------- --GGCCTCA-
KT932087        CCTTTGTGAA CCAAAA-AA- CACTTTCGCT TCGGT-AGCT G--------- --GGCCCTAA
KT932088        CCTTTGTGAA CCAAAA-AA- -CCTTTCGCT TCGGC-AGCT G--------- --GGTCCTAA
AY944137        CCTCTGTGAA CCAAAA-AA- -CCTTTCGCT TCGGC-AGCC G--------C CCGGCCAGAG
AY965758        CCTTTGTGAA CCAAAA-AA- -CCTTTCGCT TCGGC-AGCA G--------C TCGGTTGGAA
FJ380936        CTTTTGTGAA CCAAAC-CTT TCTTTTCGCT TCGGC-AGCA G-------CG GCGATCC---
KJ938573        CCTTTGTGAA CCAAAA-CAA ACCTTTCGCT TCGGC-AGCT G-------GG CCCCGTTTGG
AY773460        CCATTGTGAA CCAAATTTA- -CCTTTCGCT TCGGC-AGCG G--------- --------GG
AF106523        CCTTTGTGAA CCAAAA-AA- -CCTTTCGCT TCGGC-AGCT G--------- ----------
DQ999821        CCTTTGTGAA CCAAAA-AC- -CTTTTCGCT TCGGC-AGCA G--------C CCGGCCCGT-
U51949          CCTTTGTGAA CCAAAA-AA- CCTTTTCGCT TCGGC-AGCT G--------- --GGCCTGA-
U51945          CCTTTGTGAA CCAAAA-AA- CCTTTTCGCT TCGGC-AGCC A--------- --GGCCTCA-
AF106538        CCTTTGTGAA CCAAAA-AA- CCTTTTCGCT TCGGTTAGCG G-------GT TCGGTGGTAA

                ....|....| ....|....| ....|....| ....|....| ....|....| ....|....|
                      65         75         85         95        105        115
KT932055        AC-----CAG -CCCGTCAGC CTGCCGCTAG CACCAAACAA AAAAAACTTG -TTGTA-AAA
KT932056        AC-CGGGTTG -CCTGTCAGC CTGCCGCTAG CACCAACCA- --AAAACCTG -TTGTC-AAA
KT932057        -------CCG -CCTGTCAGC CTGCCGCTAG CACCCAACC- AAAAAACCTG -TTGTC-AAA
KT932058        -------CCG -CCTGTCAGC CTGCCGCTAG CACCCAACC- AAAAAACCTG -TTGTC-AAA
KT932059        AC--GACCCC -GGCGTCAGC CCGCCGTTAG CACCAACAT- CAAAAACTTG -TATTCTCGA
KT932060        AC--GACCCC -GGCGTCAGC CCGTCGTTAG CACCAACAT- CAAAAACTTG -TATTCTCGA
KT932061        TTCTGACCCG ---CGTGAGC CTGCCGCTGG CACCCATTT- TACAAACTTG CTCAG--AAA
KT932062        ---TCCTCCG -GACGTCAGC CTGCCGCTAG CACCAAAAA- CATAAACTTG -CAGT--AAA
KT932063        AC--AGCCTC -TGCTTCAGC CTGCCGGTAG CACCAATCA- TCAAAACTTG -CAGTTAATA
KT932064        AT--GGCCTG -GCTGTGAGC CTGCCGATGG CACCAATTT- ---AAACCTG -TCGTC--AA
KT932065        CC---GGTTG -CCTGTCAGC CTGCCGCTAG CACCCAACCA AAAAAACCTG -TTGTC--AA
KT932066        ---TCCTCCG -GACGTCAGC CTGCCGCTAG CACCAAAAA- CATAAACTTG -CAGT--AAA
KT932067        TCCCGCACGG -GACGTCAGC CTGCCGGCAG CACCCCAAA- CAAAAACTTG -CAGT--AAA
KT932068        C------CGG -TCCGTCAGC CTGCCGCTAG CACCCCTTC- -ATAAACCTG TTTGTC-AAA
KT932069        -------CCG -CCTGTCAGC CTGCCGCTAG CACCCAACC- AAAAAACCTG -TTGTC-AAA
KT932070        -------CCG -CCTGTCAGC CTGCCGCTAG CACCCAACC- -AAAAACCTG -TTGTA-AAA
KT932071        ACCCAACCAA TTCCGTAAGC CTGCCGGCAG CACCAAAAT- -TTAAACCTG -TCGTC--AA
KT932072        CA------GA -CCTGTCAGC CTGCCGCTAG CACCTT---- CAAAAACTTG -TTGTC-AAA
```

```
KT932073      --------CA -GCTGTAAGC CTGCCGATGG CACCAATTT- --AAACTTTG TTTGTA-AAA
KT932074      C------CGG -TCCGTCAGC CTGCCGCTAG CACCCCTTA- -ATAAACTTG TTTGTC-AAA
KT932075      -------CGC -TCCGTCAGC CTGCCGACAG CACCAACTT- --CAAACTTG -CAGCTTATT
KT932076      ---TCCTCCG -GACGTCAGC CTGCCGCTAG CACCAAAAA- CATAAACTTG -CAGT--AAA
KT932077      -------CCG -CCTGTCAGC CTGCCGCTAG CACCCAACC- AAAAAACCTG -TTGTC-AAA
KT932078      AC--AGCCTC -TGCGTCAGC CTGCCGGTAG CACCAATTA- -CAAAACTTG -CAGTCAAGA
KT932079      CCTTTCCGGC -CGTGTCAGC CTGCCGTTAG CACTCATCA- -AAAAACCTG -CCTT----A
KT932080      -------CGC -TCCGTCAGC CTGCCGACAG CACCAACTT- --CAAACTTG -CAGCTTATT
KT932081      -------CCG -CCTGTCAGC CTGCCGCTAG CACCCAACC- AAAAAACCTG -TTGTC-AAA
KT932082      AC--GACCCG -GTTGTCAGC CTGCCGCTAG CACCAACCT- TCAAAACTTG -CAGTT--AA
KT932083      AATGCTCTTT -GCTGTGAGC CTGCCGATGG CACCAATTT- ---AAACCTG -TCGTC--AA
KT932084      AC--GGCCCG -GTTGTCAGC CTGCCGCTAG CACAACCCC- TCAAAACTTG -CAGTT--GA
KT932085      CC--GGGTGG -CCTGTCAGC CTGCCGCTAG CACCAAAAA- -AAAAACTTG -TTGTCAAAA
KT932086      -------CCG -CCTGTCAGC CTGCCGCTAG CACCCAACC- AAAAAACCTG -TTGTC-AAA
KT932087      C------CGG -TCTGTCAGC CTGCCGCTAG CACCCCTTC- -ATAAACTTG TTTGTC-AAA
KT932088      CA------GA -CCTGTCAGC CTGCCGCTAG CACCTT---- CAAAAACTTG -TTGTC-AAA
AY944137      AT--GGCCGC -AGCTTCAGC CTGCCGGCAG CACCAAACAC AAAAAACTTG -CTTTGAAAA
AY965758      AC--AACCCC -CGCGTCAGC CTGCCGGTGG CACCAATCT- CATAAACTTG -CAGTTAAGA
FJ380936      -------CGC -CGCGTCAGC CTGCCGCTAG CACCGT---- TGAAAACTTG -CTGT----A
KJ938573      GA-------- -CCCGTCAGC CTGCCGCTAG CACCAAACA- AAAAAACTTG -TTGTC-AAA
AY773460      CTGCTTCAGC -CTTGTAAGC CTGCCGGTGG CATCATCTT- ---AAACCTG -TGTTT---A
AF106523      ---TCCTCTG -GACGTCAGC CTGCCGCTAG CACCAAAAA- CATAAACTTG -CAGT--AAA
DQ999821      ---CAGTCAA -CGCGTGAGC CTGCCGGCAG CACCAACTT- TACAAACTTG -CAGTA-ACG
U51949        -------CCG -CCTGTCAGC CTGCCGCTAG CACCAAACC- -ACAAACCTG -TTGTC-AAA
U51945        -------CCG -CCTGTCAGC CTGCCGCTAG CACCCAAAC- CACAAACCTG -TTGTC-AAA
AF106538      AA--AACCGC -TCCGTAAGC CTGCCGACAG CACCTTCTT- TGAAAACCTG -TCGT--ATA

              ....|....| ....|....| ....|....| ....|....| ....|....| ....|....|
                   125        135        145        155        165        175
KT932055      ACA-TTGTCT G-ACAACC-A AATTTTCGAA TG-AAAATCA A-AACTTTCA ACAACGGATC
KT932056      ACA-TTGTCT G-ATAACCAA AATTTTCGAA TG-AAAATCA A-AACTTTCA ACAACGGATC
KT932057      ACATTTGTCT G-ATAACCAA AATTTTCGAA TG-AAAATCA A-AACTTTCA ACAACGGATC
KT932058      ACATTTGTCT G-ATAACCAA AATTTTCGAA TG-AAAATCA A-AACTTTCA ACAACGGATC
KT932059      ACA-TTGTCT G-ATGACC-A AATTTTCGAA TG-AAAATCA A-AACTTTCA ACAACGGATC
KT932060      ACA-TTGTCT G-ATGACC-A AATTTTCGAA TG-AAAATCA A-AACTTTCA ACAACGGATC
KT932061      ACT-ATGTCT G-ATAACC-A AATTTTCGAA TG-AAAATCA A-AACTTTCA ACAACGGATC
KT932062      ACA-CTGTCT G-AT-ACC-A AATTTTCGAA TG-AAAATTA A-AACTTTCA ACAACGGATC
KT932063      ACA-TTGTCT G-ATTACC-A AATTTTCGAA TG-AAAATCA A-AACTTTCA ACAACGGATC
KT932064      ATA-TTGTCT GAATAAAC-- CATTTTCGAA TG-AAAATTA A-AACTTTCA ACAACGGATC
KT932065      ACA-TTGTCT G-ATAACC-A AATTTTCGAA TG-AAAATTA A-AACTTTCA ACAACGGATC
KT932066      ACA-CTGTCT G-AT-ACC-A AATTTTCGAA TG-AAAATTA A-AACTTTCA ACAACGGATC
KT932067      ACA-CTGTCT G-AT-ACC-A AATTTTCGAA TG-AAAATTA A-AACTTTCA ACAACGGATC
KT932068      ACA-TTGTCT G-ATAACC-A AATTTTCGAA TG-AAAATCA A-AACTTTCA ACAACGGATC
KT932069      ACA-TTGTCT G-ATAACCAA AATTTTCGAA TG-AAAATCA A-AACTTTCA ACAACGGATC
KT932070      ACA-TTGTCT G-ATAACC-A AATTTTCGAA TG-AAAATCA A-AACTTTCA ACAACGGATC
KT932071      ATA-TTGTCT G-AATACC-A TATTTTCGAA TG-AAAATTA A-AACTTTCA ACAACGGATC
KT932072      ACA-TTGTCT G-ATAACC-A AATTTTCGAA TG-AAAATTA A-AACTTTCA ACAACGGATC
KT932073      ATA-TTGTCT GAACAAAC-- CATTTTCGAA TG-AAAATTA A-AACTTTCA ACAACGGATC
KT932074      ACA-TTGTCT G-ATAACC-A AATTTTCGAA TG-AAAATCA A-AACTTTCA ACAACGGATC
KT932075      A----TGTCT G---AATCAA ATTTTTTGAA TTAAAAATCA A-AACTTTCA ACAACGGATC
KT932076      ACA-CTGTCT G-AT-ACC-A AATTTTCGAA TG-AAAATTA A-AACTTTCA ACAACGGATC
KT932077      ACATTTGTCT G-ATAACCAA AATTTTCGAA TG-AAAATCA A-AACTTTCA ACAACGGATC
KT932078      ACA-TTGTCT G-ATTACC-A AATTTTCGAA TG-AAAATCA A-AACTTTCA ACAACGGATC
KT932079      TTATATGTCT G----AACAA AAATTTTGAA TT--AAATCA A-AACTTTCA ACAACGGATC
KT932080      A----TGTCT G---AATCAA ATTTTTTGAA TTAAAAATCA ATTTCTTTCA ACAACGGATC
KT932081      ACATTTGTCT G-ATAACC-A AATTTTCGAA TG-AAAATCA A-AACTTTCA ACAACGGATC
KT932082      ACA-ATGTCT G-ACAACC-A AATTTTCGAA TG-AAAATTA A-AACTTTCA ACAACGGATC
KT932083      ATA-TTGTCT GAACAAAC-- CATTTTCGAA TG-AAAATTA A-AACTTTCA ACAACGGATC
KT932084      ACA-ATGTCT G-ACAACC-A AATTTTCGAA TG-AAAATTA A-AACTTTCA ACAACGGATC
KT932085      ACA-TTGTCT G-ATAACCAA AATTTTCGAA TG-AAAATCA A-AACTTTCA ACAACGGATC
KT932086      ACATTTGTCT G-ATAACCAA AATTTTCGAA TG-AAAATCA A-AACTTTCA ACAACGGATC
KT932087      ACA-TTGTCT G-ATAACC-A AATTTTCGAA TG-AAAATCA A-AACTTTCA ACAACGGATC
KT932088      ACA-TTGTCT G-ATAACC-A AATTTTCGAA TG-AAAATTA A-AACTTTCA ACAACGGATC
AY944137      TTA--TGTCT G-ATAACC-A AATTTTCGAA TG-AAAATTA A-AACTTTCA ACAACGGATC
AY965758      ACA-TTGTCT G-AACACC-A AATTTTCGAA TG-AAAATCA A-AACTTTCA ACAACGGATC
FJ380936      TCTCATGTCT G-AATACG-A ATATTTTGAA TT--CAATGA A-AACTTTCA ACAACGGATC
KJ938573      ACA-TTGTCT G-ATAACCAA AATTTTCGAA TG-AAAATCA A-AACTTTCA ACAACGGATC
AY773460      ATT-ATGTCT GAACAAT--A AGATTTTGAA TT--AAATCA A-AACTTTCA ACAACGGATC
AF106523      ACA-CTGTCT G-AT-ACC-A AATTTTCGAA TG-AAAATTA A-AACTTTCA ACAACGGATC
```

```
DQ999821      AAA-TTGTCT G-ACAACC-A AATTTTCGAA TG-AAAATCA A-AACTTTCA ACAACGGATC
U51949        ACA-TTGTCT G-ATAACC-A AATTTTCGAA TG-AAAATTA A-AACTTTCA ACAACGGATC
U51945        ACA-TTGTCT G-ATAACC-A AATTTTCGAA TG-AAAATTA A-AACTATCA ACAACGGA-C
AF106538      ACA-TTGTCT GAACTACC-A AATTTTCGAA TG-AAAATTA A-AACTTTCA ACAACGGATC

              ....|....| ....|....| ....|....| ....|....| ....|....| ....|....|
                  185        195        205        215        225        235
KT932055      TCTTGGTTCC CGCATCGATG AAGAACGCAG CGAAACGCGA TAGTTAATGT GAA-TTGCAG
KT932056      TCTTGGTTCC CGCATCGATG AAGAACGCAG CGAAACGCGA TAGTTAATGT GAA-TTGCAG
KT932057      TCTTGGTTCC CGCATCGATG AAGAACGCAG CGAAACGCGA TAGTTAATGT GAA-TTGCAG
KT932058      TCTTGGTTCC CGCATCGATG AAGAACGCAG CGAAACGCGA TAGTTAATGT GAA-TTGCAG
KT932059      TCTTGGTTCC CGCATCGATG AAGAACGCAG CGAAACGCGA TAGTTAATGT GAA-TTGCAG
KT932060      TCTTGGTTCC CGCATCGATG AAGAACGCAG CGAAACGCGA TAGTTAATGT GAA-TTGCAG
KT932061      TCTTGGTTCC CGCATCGATG AAGAACGCAG CGAAACGCGA TAGTTAATGT GAA-TTGCAG
KT932062      TCTTGGTTCC CGCATCGATG AAGAACGCAG CGAAACGCGA TAGTTAATGT GAA-TTGCAG
KT932063      TCTTGGTTCC CGCATCGATG AAGAACGCAG CGAAACGCGA TAGTTAATGT GAA-TTGCAG
KT932064      TCTTGGTTCT CGCATCGATG AAGAACGCAG CGAAACGCGA TAGTTAATGT GAA-TTGCAG
KT932065      TCTTGGTTCC CGCATCGATG AAGAACGCAG CGAAACGCGA TAGTTAATGT GAA-TTGCAG
KT932066      TCTTGGTTCC CGCATCGATG AAGAACGCAG CGAAACGCGA TAGTTAATGT GAA-TTGCAG
KT932067      TCTTGGTTCC CGCATCGATG AAGAACGCAG CGAAACGCGA TAGTTAATGT GAA-TTGCAG
KT932068      TCTTGGTTCC CGCATCGATG AAGAACGCAG CGAAACGCGA TAGTTAATGT GAA-TTGCAG
KT932069      TCTTGGTTCC CGCATCGATG AAGAACGCAG CGAAACGCGA TAGTTAATGT GAA-TTGCAG
KT932070      TCTTGGTTCC CGCATCGATG AAGAACGCAG CGAAACGCGA TAGTTAATGT GAA-TTGCAG
KT932071      TCTTGGTTCT CGCATCGATG AAGAACGCAG CGAAACGCGA TAGTTAATGT GAA-TTGCAG
KT932072      TCTTGGTTCC CGCATCGATG AAGAACGCAG CGAAACGCGA TAGTTAATGT GAA-TTGCAG
KT932073      TCTTGGTTCT CGCATCGATG AAGAACGCAG CGAAACGCGA TAGTTAATGT GAA-TTGCAG
KT932074      TCTTGGTTCC CGCATCGATG AAGAACGCAG CGAAACGCGA TAGTTAATGT GAA-TTGCAG
KT932075      TCTTGGTTCC CGCATCGATG AAGAACGCAG CGAAACGCGA TAGTTAATGT GAA-TTGCAG
KT932076      TCTTGGTTCC CGCATCGATG AAGAACGCAG GGAAACGCGA TAGTTAATGT GAA-TTGCAG
KT932077      TCTTGGTTCC CGCATCGATG AAGAACGCAG CGAAACGCGA TAGTTAATGT GAA-TTGCAG
KT932078      TCTTGGTTCC CGCATCGATG AAGAACGCAG CGAAACGCGA TAGTTAATGT GAA-TTGCAG
KT932079      TCTTGGTTCC CGCATCGATG AAGAACGCAG CGAAACGCGA TAGTTAATGT GAA-TTGCAG
KT932080      TCTTGGTTCC CGCATCGATG AAGAACGCAG CGAAACGCGA TAGTTAATGT GAA-TTGCAG
KT932081      TCTTGGTTCC CGCATCGATG AAGAACGCAG CGAAACGCGA TAGTTAATGT GAA-TTGCAG
KT932082      TCTTGGTTCC CGCATCGATG AAGAACGCAG CGAAACGCGA TAGTTAATGT GAA-TTGCAG
KT932083      TCTTGGTTCT CGCATCGATG AAGAACGCAG CGAAACGCGA TAGTTAATGT GAA-TTGCAG
KT932084      TCTTGGTTCC CGCATCGATG AAGAACGCAG CGAAACGCGA TAGTTAATGT GAA-TTGCAG
KT932085      TCTTGGTTCC CGCATCGATG AAGAACGCAG CGAAACGCGA TAGTTAATGT GAA-TTGCAG
KT932086      TCTTGGTTCC CGCATCGATG AAGAACGCAG CGAAACGCGA TAGTTAATGT GAA-TTGCAG
KT932087      TCTTGGTTCC CGCATCGATG AAGAACGCAG CGAAACGCGA TAGTTAATGT GAA-TTGCAG
KT932088      TCTTGGTTCC CGCATCGATG AAGAACGCAG CGAAACGCGA TAGTTAATGT GAA-TTGCAG
AY944137      TCTTGGTTCC CGCATCGATG AAGAACGCAG CGAAACGCGA TAGTTAATGT GAA-TTGCAG
AY965758      TCTTGGTTCC CGCATCGATG AAGAACGCAG CGAAACGCGA TAGTTAATGT GAA-TTGCAG
FJ380936      TCTTGGTTCC CGCATCGATG AAGAACGCAG CGAAACGCGA TAGTTAATGT GAA-TTGCAG
KJ938573      TCTTGGTTCC CGCATCGATG AAGAACGCAG CGAAACGCGA TAGTTAATGT GAA-TTGCAG
AY773460      TCTTGGTTCC CGCATCGATG AAGAACGCAG CGAAACGCGA TAGTTAATGT GAA-TTGCAG
AF106523      TCTTGGTTCC CGCATCGATG AAGAACGCAG CGAAACGCGA TAGTTAATGT GAA-TTGCAG
DQ999821      TCTTGGTTCC CGCATCGATG AAGAACGCAG CGAAACGCGA TAGTTAATGT GAA-TTGCAG
U51949        TCTTGGTTCC CGCATCGATG AAGAACGCAG CGAAACGCGA TAGTTAATGT GAA-TTGCAG
U51945        TCTTGGTTCC CGCATCGATG AAGAACGCAG CGAAACGCGA TAGTTAATGT GAATTTGCAG
AF106538      TCTTGGTTCT CGCATCGATG AAGAACGCAG CGAAACGCGA TAGTTAATGT GAA-TTGCAG

              ....|....| ....|....| ....|....| ....|....| ....|....| ....|....|
                  245        255        265        275        285        295
KT932055      AATTCAGTGA ATCATCGAGT CTTTGAACGC ACATTGCGCC CATTGGTATT CCTTTGGGCA
KT932056      AATTCAGTGA ATCATCGAGT CTTTGAACGC ACATTGCGCC CATTGGTATT CCTTTGGGCA
KT932057      AATTCAGTGA ATCATCGAGT CTTTGAACGC ACATTGCGCC CATTGGTATT CCTTTGGGCA
KT932058      AATTCAGTGA ATCATCGAGT CTTTGAACGC ACATTGCGCC CATTGGTATT CCTTTGGGCA
KT932059      AATTCAGTGA ATCATCGAGT CTTTGAACGC ACATTGCGCC CATTGGTATT CCATTGGGCA
KT932060      AATTCAGTGA ATCATCGAGT CTTTGAACGC ACATTGCGCC CATTGGTATT CCATTGGGCA
KT932061      AATTCAGTGA ATCATCGAGT CTTTGAACGC ACATTGCGCC CATCGGTATT CCGTTGGGCA
KT932062      AATTCAGTGA ATCATCGAGT CTTTGAACGC ACATTGCGCT CACCGGTATT CCGGTGAGCA
KT932063      AATTCAGTGA ATCATCGAGT CTTTGAACGC ACATTGCGCC CATTGGTATT CCATTGGGCA
KT932064      AATTCAGTGA ATCATCGAGT CTTTGAACGC ACATTGCGCC CATCGGTATT CCTTTGGGCA
KT932065      AATTCAGTGA ATCATCGAGT CTTTGAACGC ACATTGCGCC CATTGGTATT CCTTTGGGCA
KT932066      AATTCAGTGA ATCATCGAGT CTTTGAACGC ACATTGCGCT CACCGGTATT CCGGTGAGCA
KT932067      AATTCAGTGA ATCATCGAGT CTTTGAACGC ACATTGCGCC CACCGGTATT CCGGTGGGCA
KT932068      AATTCAGTGA ATCATCGAGT CTTTGAACGC ACATTGCGCC CATTGGTATT CCTTTGGGCA
KT932069      AATTCAGTGA ATCATCGAGT CTTTGAACGC ACATTGCGCC CATTGGTATT CCTTTGGGCA
```

```
KT932070        AATTCAGTGA ATCATCGAGT CTTTGAACGC ACATTGCGCC CATTGGTATT CCTTTGGGCA
KT932071        AATTCAGTGA ATCATCGAGT CTTTGAACGC ACATTGCGCC TATTGGTATT CCATTAGGCA
KT932072        AATTCAGTGA ATCATCGAGT CTTTGAACGC ACATTGCGCC CATTGGTATT CCTTTGGGCA
KT932073        AATTCAGTGA ATCATCGAGT CTTTGAACGC ACATTGCGCC CATCGGTATT CCTTTGGGCA
KT932074        AATTCAGTGA ATCATCGAGT CTTTGAACGC ACATTGCGCC CATTGGTATT CCTTTGGGCA
KT932075        AATTCAGTGA ATCATCGAGT CTTTGAACGC ACATTGCGCC CATTGGTATT CCATTGGGCA
KT932076        AATTCAGTGA ATCATCGAGT CTTTGAACGC ACATTGCGCT CACCGGTATT CCGGTGAGCA
KT932077        AATTCAGTGA ATCATCGAGT CTTTGAACGC ACATTGCGCC CATTGGTATT CCTTTGGGCA
KT932078        AATTCAGTGA ATCATCGAGT CTTTGAACGC ACATTGCGCC CATTGGTATT CCATTGGGCA
KT932079        AATTCAGTGA ATCATCGAGT CTTTGAACGC ATATTGCGCC CATTGGTATT CCATTGGGCA
KT932080        AATTCAGTGA ATCATCGAGT CTTTGAACGC ACATTGCGCC CATTGGTATT CCATTGGGCA
KT932081        AATTCAGTGA ATCATCGAGT CTTTGAACGC ACATTGCGCC CATTGGTATT CCTTTGGGCA
KT932082        AATTCAGTGA ATCATCGAGT CTTTGAACGC ACATTGCGCC CATTGGTATT CCATTGGGCA
KT932083        AATTCAGTGA ATCATCGAGT CTTTGAACGC ACATTGCGCC CATCGGTATT CCTTTGGGCA
KT932084        AATTCAGTGA ATCATCGAGT CTTTGAACGC ACATTGCGCC CATTGGTATT CCATTGGGCA
KT932085        AATTCAGTGA ATCATCGAGT CTTTGAACGC ACATTGCGCC CATTGGTATT CCTTTGGGCA
KT932086        AATTCAGTGA ATCATCGAGT CTTTGAACGC ACATTGCGCC CATTGGTATT CCTTTGGGCA
KT932087        AATTCAGTGA ATCATCGAGT CTTTGAACGC ACATTGCGCC CATTGGTATT CCTTTGGGCA
KT932088        AATTCAGTGA ATCATCGAGT CTTTGAACGC ACATTGCGCC CATTGGTATT CCTTTGGGCA
AY944137        AATTCAGTGA ATCATCGAAT CTTTGAACGC ACATTGCGCC CATCGGTATT CCGTTGGGCA
AY965758        AATTCAGTGA ATCATCGAGT CTTTGAACGC ACATTGCGCC CATTGGTATT CCATTGGGCA
FJ380936        AATTCAGTGA ATCATCGAGT CTTTGAACGC ATATTGCGCC CATTGGTATT CCATTGGGCA
KJ938573        AATTCAGTGA ATCATCGAGT CTTTGAACGC ACATTGCGCC CATTGGTATT CCTTTGGGCA
AY773460        AATTCAGTGA ATCATCGAGT CTTTGAACGC ACATTGCGCC CATTGGTATT CCATTGGGCA
AF106523        AATTCAGTGA ATCATCGAGT CTTTGAACGC ACATTGCGCT CACCGGTATT CCGGTGAGCA
DQ999821        AATTCAGTGA ATCATCGAGT CTTTGAACGC ACATTGCGCC CATCGGTATT CCGTTGGGCA
U51949          AATTCAGTGA ATCATCGAGT CTTTGAACGC ACATTGCGCC CATTGGTATT CCTTTGGGCA
U51945          AATTCAGTGA ATCATCGAGT CTTTGAACGC ACATTGCGCC CATTGGTATT CCTTTGGGCA
AF106538        AATTCAGTGA ATCATCGAGT CTTTGAACGC ACATTGCGCC CATAGGTATT CCTTTGGGCA

                ....|....| ....|....| ....|....| ....|....| ....|....| ....|....|
                    305        315        325        335        345        355
KT932055        TGTCTGTTTG AGCGTCATTA CAA-CCCTCA GCTAACCGCT GG-TTTTGGA CCCG----AA
KT932056        TGTCTGTTTG AGCGTCATTA CAA-CCCTCA GCTAACCGCT GG-TTTTGAA CCGG----AA
KT932057        TGTCTGTTTG AGCGTCATTA CAACCCCTCA GC-TAACGCT GG-TTTTGAA CCGG----GA
KT932058        TGTCTGTTTG AGCGTCATTA CAACCCCTCA GC-TAACGCT GG-TTTTGAA CCGG----GA
KT932059        TGTCTGTTTG AGCGTCATTA CAA-CCCTCG GT-CACCACC GG-TTTTGAG CAAG------
KT932060        TGTCTGTTTG AGCGTCATTA CAA-CCCTCG GT-CACCACC GG-TTTTGAG CAAG------
KT932061        TGTCTGTTTG AGCGTCATCA CAA-CCCTCG AC-ACCTGTC GG-TTATGAG CCCG----CC
KT932062        TGTCTGTCTG AGCGTCATTA CAA-CCCTCG GT-CCACACC GG-TTTTGAG CGAG---CGC
KT932063        TGTCTGTTTG AGCGTCATTA CAA-CCCTCG GT-CACCACC GG-TTTTGAG CAAG------
KT932064        TGTCTGTTTG AGCGTCATTA CAAACCCTCA GC-GCAAGCT GG-TTTTGAG TTGTGAGGAA
KT932065        TGTCTGTTTG AGCGTCATTA CAACCCCTCA GC-TACCGCT GG-TTTTGAA TCGG----AA
KT932066        TGTCTGTCTG AGCGTCATTA CAA-CCCTCG GT-CCACACC GG-TTTTGAG CGAG---CGC
KT932067        TGTCTGTTTG AGCGTCATTA CAA-CCCTCG GT-CCACACC GG-TTTTGAG CGAG----CG
KT932068        TGTCTGTTTG AGCGTCATTA CAG-CCCTCA GCTAACCGCT GG-TTTTGAA CCTG----AA
KT932069        TGTCTGTTTG AGCGTCATTA CAACCCCTCA GC-TAACGCT GG-TTTTGAA CCGG----GA
KT932070        TGTCTGTTTG AGCGTCATTA CAACCCCTCA GC-TAACGCT GG-TTTTGAA CCGG----GA
KT932071        TGTCTGTTTG AGCGTCATTA CAACCCCTCA GC-GCAAGCT GG-TTTTGAG CCGG------
KT932072        TGTCTGTTTG AGCGTCATTA CAACCCCTCA GC-TAACGCT GG-TTTTGAA CCCG----AA
KT932073        TGTCTGTTTG AGCGTCATTA CAACACCTCA GC-GCAAGCT GG-TTTTGAG CTGT----AA
KT932074        TGTCTGTTTG AGCGTCATTA CAA-CCCTCA GCTAACCGCT GG-TTTTGAA CCTG----AA
KT932075        TGTCTGTTTG AGCGTCATTA CAAACCTTTG AC-ACCAGTC AG-TATTGGG CCGG-CTGCC
KT932076        TGTCTGTCTG AGCGTCATTA CAA-CCCTCG GT-CCACACC GG-TTTTGAG CGAG---CGC
KT932077        TGTCTGTTTG AGCGTCATTA CAACCCCTCA GC-TAACGCT GG-TTTTGAA CCGG----GA
KT932078        TGTCTGTTTG AGCGTCATTA CAA-CCCTCG GT-CACCACC GG-TCTTGAG CAAG------
KT932079        TGTCTGTTTG AGCGTCATTA CAAACCCTCG AC-TTTTGTC GG-TTTTGAG CTGG-CTTTA
KT932080        TGTCTGTTTG AGCGTCATTA CAAACCTTTG AC-ACCAGTC AG-TATTGGG CCGG-CTGCC
KT932081        TGTCTGTTTG AGCGTCATTA CAACCCCTCA GC-TAACGCT GG-TTTTGAA CCGG----GA
KT932082        TGTCTGTTTG AGCGTCATTA CAA-CCCTCG GT-ACCAACC GG-TTTTGAG CAAG-----C
KT932083        TGTCTGTTTG AGCGTCATTA CAAACCCTCA GC-GAAAGCT GG-TTTTGAG TTGTGAGGAA
KT932084        TGTCTGTTTG AGCGTCATTA CAA-CCCTCG GT-TCCAACC GG-TTTTGAG CGTG----CC
KT932085        TGTCTGTTTG AGCGTCATTA CAACCCCTCA GC-TAACGCT GG-TTTTGAA CCCG----AA
KT932086        TGTCTGTTTG AGCGTCATTA CAACCCCTCA GC-TAACGCT GG-TTTTGAA CCGG----GA
KT932087        TGTCTGTTTG AGCGTCATTA CAA-CCCTCA GCTAACCGCT GGTTTTTGAA CCTG----AA
KT932088        TGTCTGTTTG AGCGTCATTA CAACCCCTCA GC-TAACGCT GG-TTTTGAA CCCG----AA
AY944137        TGTCTGTTTG AGCGTCATTA CAA-CCCTCG AC-ACCTGTC GG-TTCTGAG CCCG---GAA
AY965758        TGTCTGTTTG AGCGTCATTA CAA-CCCTCG GT-CACCACC GG-TTTTGAG CGAG------
FJ380936        TGTCTGTTTG AGCGTCATTT CAAACCCTCG AC-TTTGGTC GG-TATTGAG CTGG-CTTTA
```

```
KJ938573     TGTCTGTTTG AGCGTCATTA CAA-CCCTCA GCTACCCGCT GG-TTTTGAA CCCG----AA
AY773460     TGTCTGTTTG AGCGTCATTA CATACCCTCG AC-ACCCGTC GG-TATTGGG CTGG-CTTTA
AF106523     TGTCTGTCTG AGCGTCATTA CAA-CCCTCG GT-CCACACC GG-TTTTGAG CGAG---CGC
DQ999821     TGTCTGTTTG AGCGTCATTA CAA-CCCTCG AC-AACCGTC GG-TTATGAG CCCG---CCC
U51949       TGTCTGTTTG AGCGTCATTA CAA-CCCTCA GCTAACCGCT GG-TTTTGAA CCGG----AA
U51945       TGTCTGTTTG AGCGTCATTA CAA-CCCTCA GCTAACCGCT GG-TTTTGAA CCGG----AA
AF106538     TGTCTGTTTG AGCGTCATTA CAACCCCTCA GC-GAAAGCT GG-TTTTGAA CGGG----AG

             ....|....| ....|....| ....|....| ....|....| ....|....| ....|....|
                 365        375        385        395        405        415
KT932055     CGGCTTGA-- --------CT GCCGCG---C AGGTTTTAAA GTTGTAAGCT CTGC-TGGCT
KT932056     CGGGTCAC-- ---------- ACCGCG---C CGGTTTTAAA GTTGTAAGCT CTGC-TGGCC
KT932057     CAGGTTAA-- --------CA CCCGCA---C CGGTTTTAAA GTTGTAAGCT CTGC-TGGCT
KT932058     CAGGTTAA-- --------CA CCCGCA---C CGGTTTTAAA GTTGTAAGCT CTGC-TGGCT
KT932059     CGGGGACCCC TCG-----GG GCCCGG---C CGGCTTTAAA GTTGTAAGCT CTGCTTGGCC
KT932060     CGGGGACCCC TCG-----GG GCCCGG---C CGGCTTTAAA GTTGTAAGCT CTGC-TGGCC
KT932061     TGGCGTGA-- --------AA CCCGGG---C CGGCTTCAAA TTTGTGAGCT CTGC-TGGCT
KT932062     CTGGGTCTCA C-------CA CCCGAG---C TGGCTTTAAA GTTGTAAGCT CTGC-TGGCT
KT932063     CGAGGTCTCC --------GG ACCCGG---C TGGCTTTAAA GTTGTAAGCT CTGC-TGGCT
KT932064     AAATGCCCCT TGG-----GG CACGCACCTC TCGCTTTAAA GTTGTACGCT CTGC-TGACC
KT932065     CGGGTCAC-- ---------- ACCGCG---C CGGTTTTAAA GTTGTAAGCT CTGC-TGGCC
KT932066     CTGGGTCTCA C-------CA CCCGAG---C TGGCTTTAAA GTTGTAAGCT CTGC-TGGCT
KT932067     CTGGGTCCTC TGG-----GA CCCGAG---C TGGCTTTAAA GTTGTAAGCT CTGC-TGGCT
KT932068     CGGGGTAA-- --------CA CCTGCG---C TGGTTTTAAA GTTGTAAGCT CTGC-TGGCC
KT932069     CAGGTTAA-- --------CA CCCGCA---C CGGTTTTAAA GTTGTAAGCT CTGC-TGGCT
KT932070     CAGGATCA-- --------CA CCCGCA---C CGGTTTTAAA GTTGTAAGCT CTGC-TGGCT
KT932071     TTGGTCTTGA A-------AA AGCCAA---C CGGTTTTAAA GTTGTAAGCT CTGC-TGGCT
KT932072     CGGTTTCC-- --------AC GCCGTG---C CGGTTTTAAA GTTGTAAGCT CTGC-TGGCC
KT932073     GGGTTCGCTT CGGC----TA CCCT-----T CCGCTTTAAA GTTGTAAGCT CTGC-TGGCT
KT932074     CGGGGTAA-- --------CA CCTGCG---C TGGTTTTAAA GTTGTAAGCT CTGC-TGGCC
KT932075     CGAGCGAA-- --------AG CTCGCG---C CGGCTTTAAA GTAGTACGCT TTGC-CAACC
KT932076     CTGGGTCTCA C-------CA CCCGAG---C TGGCTTTAAA GTTGTAAGCT CTGC-TGGCT
KT932077     CAGGTTAA-- --------CA CCCGCA---C TGGTTTTAAA GTTGTAAGCT CTGC-TGGCT
KT932078     CGAGGTCTCC --------GG ACCCAG---C TGGCTTTAAA GTTGTAAGCT CTGC-TGGCT
KT932079     TGGGTGCA-- --------AG CCCAAG---C CGGTTTTAAA GTGGTAAGCT TTGC-TGACC
KT932080     CAAGCGAA-- --------AG CTCGCG---C CGGCTTTAAA GTAGTACGCT TTGC-CAACC
KT932081     CAGGTTAAA- --------CA CCCGCA---C CGGTTTTAAA GTTGTAAGCT CTGC-TGGCT
KT932082     CAGGGTCCC- --------CG ACCCGG---C TGGCTTTAAA GTTGTAAGCT CTGC-TGGCT
KT932083     AAAATGCCCC TTGGGGCACG CACCCT---C TCGCTTTAAA GTTGTACGCT CTGC-TGACC
KT932084     CGGGTCCC-- --------CG ACCCGG---C CGGCTTTAAA GTTGTAAGCT CTGC-TGCCC
KT932085     CGGTTGAT-- ---------- GCCGCG---C CGGTTTTAAA GTTGTAAGCT CTGC-TGGCC
KT932086     CAGGTTAA-- --------CA CCCGCA---C CGGTTTTAAA GTTGTAAGCT CTGC-TGGCT
KT932087     CGGGGTAA-- --------CA CCTGCG---C TGGTTTTAAA GTTGTAAGCT CTGC-TGGCC
KT932088     CGGTTTCC-- --------AC GCCGTG---C CGGTTTTAAA GTTGTAAGCT CTGC-TGGCC
AY944137     CGGCGTAA-- --------CA CCCGCC---C CGGCTTTAAA GTTGTAAGCT CTGC-TGGCC
AY965758     CGGGGTCTTC --------GG ACCCGG---C TGGCTTTAAA GTTGTAAGCT CTGC-TGGCT
FJ380936     TGGGTGCG-- --------AA CCCAGG---C CGGTTTTAAA GTGGTAGGCT TTGC-TGTCT
KJ938573     CGGTGCCCCC TAACCGGGGA ACCGAG---C CGGTTTTAAA GTTGTAAGCT CTGC-TGGCC
AY773460     TGAATGCA-- --------AA TTCAAG---C CGGCTTTAAA GTGGCAAGCT CTGC-TGACC
AF106523     CTGGGTCTCA C-------CA CCCGAG---C TGGCTTTAAA GTTGTAAGCT CTGC-TGGCT
DQ999821     CGGCGTCA-- --------AA CCCGGG---C CGGCTTTAAA GTTGTAAGCT CTGC-TGGCT
U51949       CGGGTACC-- --------CA CCCGCA---C CGATTTTAAA GTTGTAAGCT CTGC-TGGCT
U51945       CGGGTGTC-- --------CA CCCGCA---C CGGTTTTAAA GTTGTAAGCT CTGC-TGGCT
AF106538     CTTG------ --------GA AACGAGCGGC CCGTTTTAAA GTTGTAAGCT CTGC-TGTCC

             ....|....| ....|....| ....|....| ....|....| ....|....| ....|....|
                 425        435        445        455        465        475
KT932055     -GCTCTGCCC CAAC--CGGA ACATAGTAA- --GC-ACTAC TTTTGTTAA- GGT-GAA--G
KT932056     -GCTCCGCAC CAAC--CAGA ACATAGTAA- --GC-ACTAC TTTTGTTAG- GGT-GAA--G
KT932057     -GCTCTGCCC CAAC--CGGA ACATAGTAA- -AAC-ACTAC --TTGTTAA- GGC-GAG--G
KT932058     -GCTCTGCCC CAAC--CGGA ACATAGTAA- -AAC-ACTAC --TTGTTAA- GGC-GAG--G
KT932059     -GCTCCGCCT CGCT---AGA ACATAGTAA- --AA-CCTAC --TTGTTCGC GGTCGAG-GT
KT932060     -GCTCTGCCT CGCT---AGA ACATAGTAA- --AA-CCTAC --TTGTTCGC GGTCGAG-GT
KT932061     -GCTGCGCCT GACC---GAG ACATAGTAA- -GAC--CTAC --TTGTCAAA GGT-GAG--G
KT932062     -GCCCTGCCC GACT---TGA ACATAGTAA- --AC-TTTGC --TTGTTCGC GGTTGAG--G
KT932063     -GCCAGGCCC AACC---AGA ACATAGTAA- --AATCATGC --TTGTTCAC GGT-TCGCGG
KT932064     --TTTAGCTC GGAC-TAAAA ACATAGTAA- --ATTCTTGC --TTGTTTA- -ACAGAACGG
KT932065     -GCTCTGCCC CAAC--CAGA ACATAGTAA- --GCAACTAC --TTGTTAG- GGT-GAA--G
KT932066     -GCCCTGCCC GACT---TGA ACATAGTAA- --AC-TTTGC --TTGTTCGC GGTTGAG--G
```

```
KT932067      -GCCCCGCCC GACT---TGA ACATAGTAA- --AC-TTTGC --TTGTTCGC GGTTTAG--G
KT932068      -GCTCTGCCA AACC---AGA ACATAGTAA- --GC-TCTAC --TTGTTTG- GGT-GAA--G
KT932069      -GCTCTGCCC CAAC--CGGA ACATAGTAAT CCAAAACTAC --TTGTTAA- GGC-GAG--G
KT932070      -GCTCTGCCC CAAC--CGGA ACATAGTAA- -AAACACTAC --TTGTTAA- GGC-GAG--G
KT932071      -GCTACGCCC AACC--AAAA ACATAGTAA- --ACTTTTGC --TTGTTTGA AGGTGAG--G
KT932072      -GCTCTGCCC CAAC--CAGA ACATAGTAA- --GCAACTAC --TTGTTAG- GGTGAAG---
KT932073      --TCCAGCCT GACC-G-GAA ACATAGTAA- --ATTTCTTG CCTTGTTTAT GGATAGGTTG
KT932074      -GCTCTGCCA AACC---AGA ACATAGTAA- --GC-TCTAC --TTGTTTG- GGT-GAA--G
KT932075      -GCCATTCCA AGCC--AAAA ACATAGTAA- ---TCATAAC GCTTGTTGAG GATGGAT--G
KT932076      -GCCCTGCCC GACT---TGA ACATAGTAA- --AC-TTTGC --TTGTTCGC GGTTGAG--G
KT932077      -GCTCTGCCC CAAC--CGGA ACATAGTAA- -AAC-ACTAC --TTGTTAA- GGC-GAG--G
KT932078      -GCCCGGCCC AACC---AGA ACATAGTAA- --AACAATGC --TTGTTCAC GGT-TCGCGG
KT932079      -ACTGCTCCA AACC--AAAA ACATAGTAA- --AT-TCTGC --TTGTTGAT GGTGGGGTGG
KT932080      -GCCATTCCA AGCC--AAAA ACATAGTAA- ---TCATAAC GCTTGTTGAG GATGGAT--G
KT932081      -GCTCTGCCC CAAC--CGGA ACATAGTAA- -AAACACTAC --TTGTTAA- GGC-GAG--G
KT932082      -GCCCGGCCT GACC---AGA ACATAGTAA- --AACACTAC --TTGTTCAC AGTTGAG--G
KT932083      --TTTAGCTC GGAC-TAAAA ACATAGTAA- --ATTCTTGC --TTGTTTTA CG--GAACGG
KT932084      -GCCCAGCCC GGCC---AGA ACATAGTAA- --AA-ACTAC --TTGTTCCC GGTCAGG--A
KT932085      -GCTCCGCCC CAAC--CAGA ACATAGTAA- --GC-ACTAC TTTTGTTAG- GGT-GAA--G
KT932086      -GCTCTGCCC CAAC--CGGA ACATAGTAA- -AAC-ACTAC --TTGTTAA- GGC-GAG--G
KT932087      -GCTCTGCCA AACC---AGA ACATAGTAA- --GC-TCTAC --TTGTTTG- GGT-GAA--G
KT932088      -GCTCTGCCC CAAC--CAGA ACATAGTAA- --GCAACTAC --TTGTTAG- GGTGAAG---
AY944137      -GCTCCGCCT GACC--GAAA ACATAGTAA- --AC--CTAC --TTGTTGGA GG--GA---G
AY965758      -GCCAGGCCC GACC---AGA ACATAGTAA- --AATCATGC --TTGTTCAC GGT-TAGCGG
FJ380936      -GCTGCTCCA AACC--AAAA ACATAGTAA- -----ACTGT ACTTGTTGAT GGTGGGGTGG
KJ938573      -GCTCCGCCC CAAC--CAGA ACATAGTAA- -AAC-ACTAC TTTTGTTAG- GGT-CAA--G
AY773460      -ACTGCTCCA AGTC---AAA ACATAGTAA- TAAC-AATAC --GTGTAGAA GGC-GAA--G
AF106523      -GCCCTGCCC GACT---TGA ACATAGTAA- --AC-TTTGC --TTGTTCGC GGTTGAG--G
DQ999821      --GTCCGCCT GACC---GAG ACATAGTAA- -AAC--CTAC --TTGTCGAC GG--GAC--G
U51949        -GCTCTGCCA AACC---AGA ACATAGTAA- -AAAAACTAC --TTGTTGG- GGT-GAA--G
U51945        -GCTCTGCCA AACC---AGA ACATAGTAA- --AA-ACTAC --TTGTTGG- GGT-GAA--G
AF106538      AGCCTCGCCT GACCGAAAAA ACGTAGTAA- -AACTTTTGC TCTCGTTTGA AATGGAAGGG

              ....|....| ....|....| ..
                  485        495
KT932055      CA-GAACGG- -TACGGCCTG TA
KT932056      TG-GAACGG- -TACGGCCTG TA
KT932057      CG-AAGCGG- -TACGGCCTG AA
KT932058      CG-AAGCGG- -TACGGCCTG AA
KT932059      CGGAAGCGG- -TACGGCCTG AA
KT932060      CGGAAGCGG- -TACGGCCTG AA
KT932061      CGGGGGCAG- -TTCGGCCTG AA
KT932062      TGGAAGCGG- -TACGGCCTG AA
KT932063      TCGAAGCGG- -TACGGCCTG AA
KT932064      TTGAAGGAT- ---CGGCCTG AA
KT932065      CT-GAACGG- -TACGGCCTG AA
KT932066      TGGAAGCGG- -TACGGCCTG AA
KT932067      CGGAAGCGG- -TACGGCCTG AA
KT932068      CTGGAACGGT TTACGGCCTG AA
KT932069      CG-AAGCGG- -TACGGCCTG AA
KT932070      CT-AAGCGG- -TACGGCCTG AA
KT932071      CT-TGCTGG- -ACCGGCCTG AA
KT932072      -GTGAACGG- -TATGGCCTG GA
KT932073      CGAGG----- -TTCGGCCTG AA
KT932074      CTGGAACGGT TTACGGCCTG AA
KT932075      GACTTCTGG- -TTCGGCCTG AA
KT932076      TGGAAGCGG- -TACGGCCTG AA
KT932077      CG-AAGCGG- -TACGGCCTG AA
KT932078      TCGAAGCGG- -TACGGCCTG AA
KT932079      T--------- -TCCAGCCTG GA
KT932080      GACTTCTGG- -TTCGGCCTG AA
KT932081      CG-AAGCGG- -TACGGCCTG AA
KT932082      TCGAAGCGG- -TACGGCCTG AA
KT932083      TTGGAGGAT- ---CGGCCTG AA
KT932084      TTGAAGCGG- -TGCGGCCTG AA
KT932085      CC-GAACGG- -TATGGCCTG AA
KT932086      CG-AAGCGG- -TACGGCCTG AA
KT932087      CTGGAACGGT TTACGGCCTG AA
KT932088      -GTGAACGG- -TATGGCCTG GA
```

AY944137	CGCGAACGG- -TACGGCCTG AA
AY965758	TCGAAGCGG- -TACGGCCTG AA
FJ380936	C--------- -TCCAGCCTT GA
KJ938573	CG-GAACGGT TTTCGGCCTG AA
AY773460	AGTAGTTGG- -TTCGGCCTG AA
AF106523	TGGAAGCGG- -TACGGCCTG AA
DQ999821	GCTGAACGG- -TTCGGCCTG AA
U51949	CT-AAGCGG- -TACGGCCTG GA
U51945	CT-AGGCGG- -TACGGCCTG TA
AF106538	TTGGCTTGG- -TGCGGCCTG AA

Figure S3: Phylogenetic analyses based on the ITS marker.

The tree was produced using PHYML 3.0. The reliability of the tree topology was evaluated using bootstrap support with 100 replications.

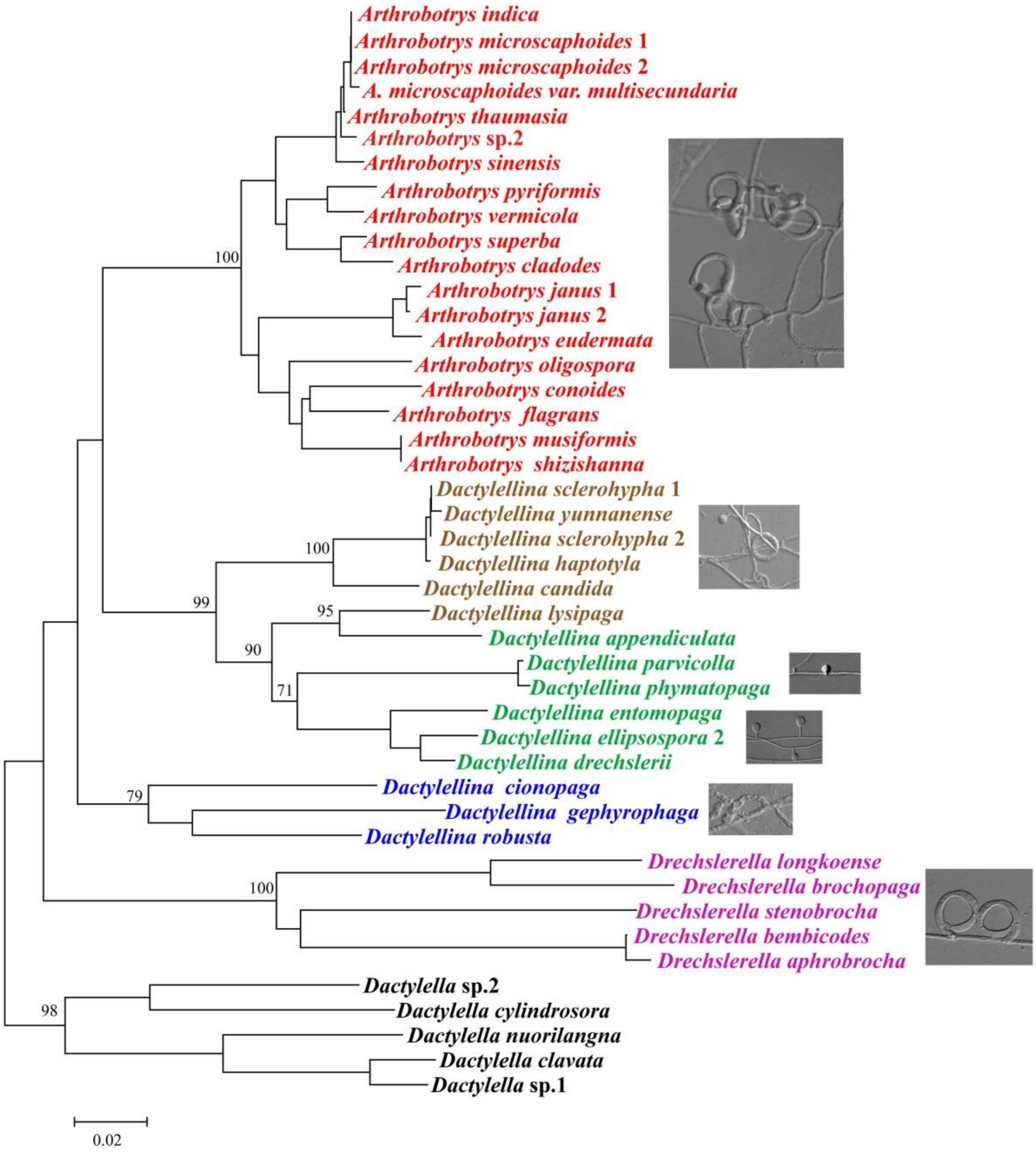

Supplement: Supplementary Information [file srep22609-s1.pdf]
